# A genome-wide association study of plasma concentrations of warfarin enantiomers and metabolites in sub-Saharan black-African patients

**DOI:** 10.3389/fphar.2022.967082

**Published:** 2022-09-23

**Authors:** Innocent G. Asiimwe, Marc Blockman, Karen Cohen, Clint Cupido, Claire Hutchinson, Barry Jacobson, Mohammed Lamorde, Jennie Morgan, Johannes P. Mouton, Doreen Nakagaayi, Emmy Okello, Elise Schapkaitz, Christine Sekaggya-Wiltshire, Jerome R. Semakula, Catriona Waitt, Eunice J. Zhang, Andrea L. Jorgensen, Munir Pirmohamed

**Affiliations:** ^1^ The Wolfson Centre for Personalized Medicine, Department of Pharmacology and Therapeutics, Institute of Systems, Molecular and Integrative Biology, University of Liverpool, Liverpool, United Kingdom; ^2^ Division of Clinical Pharmacology, Department of Medicine, University of Cape Town, Cape Town, South Africa; ^3^ Victoria Hospital Internal Medicine Research Initiative, Victoria Hospital Wynberg and Department of Medicine, University of Cape Town, Cape Town, South Africa; ^4^ Department of Molecular Medicine and Haematology, University of the Witwatersrand, Johannesburg, South Africa; ^5^ Infectious Diseases Institute, Makerere University College of Health Sciences, Kampala, Uganda; ^6^ Metro District Health Services, Western Cape Department of Health, Cape Town, South Africa; ^7^ Uganda Heart Institute, Kampala, Uganda; ^8^ Department of Molecular Medicine and Hematology, Charlotte Maxeke Johannesburg Academic Hospital National Health Laboratory System Complex and University of Witwatersrand, Johannesburg, South Africa; ^9^ Department of Health Data Science, Institute of Population Health Sciences, University of Liverpool, Liverpool, United Kingdom

**Keywords:** black-African, genome-wide association study, personalized medicine, pharmacokinetics, warfarin

## Abstract

Diversity in pharmacogenomic studies is poor, especially in relation to the inclusion of black African patients. Lack of funding and difficulties in recruitment, together with the requirement for large sample sizes because of the extensive genetic diversity in Africa, are amongst the factors which have hampered pharmacogenomic studies in Africa. Warfarin is widely used in sub-Saharan Africa, but as in other populations, dosing is highly variable due to genetic and non-genetic factors. In order to identify genetic factors determining warfarin response variability, we have conducted a genome-wide association study (GWAS) of plasma concentrations of warfarin enantiomers/metabolites in sub-Saharan black-Africans. This overcomes the issue of non-adherence and may have greater sensitivity at genome-wide level, to identify pharmacokinetic gene variants than focusing on mean weekly dose, the usual end-point used in previous studies. Participants recruited at 12 outpatient sites in Uganda and South Africa on stable warfarin dose were genotyped using the Illumina Infinium H3Africa Consortium Array v2. Imputation was conducted using the 1,000 Genomes Project phase III reference panel. Warfarin/metabolite plasma concentrations were determined by high-performance liquid chromatography with tandem mass spectrometry. Multivariable linear regression was undertaken, with adjustment made for five non-genetic covariates and ten principal components of genetic ancestry. After quality control procedures, 548 participants and 17,268,054 SNPs were retained. CYP2C9*8, CYP2C9*9, CYP2C9*11, and the *CYP2C* cluster SNP rs12777823 passed the Bonferroni-adjusted replication significance threshold (*p* < 3.21E-04) for warfarin/metabolite ratios. In an exploratory GWAS analysis, 373 unique SNPs in 13 genes, including CYP2C9*8, passed the Bonferroni-adjusted genome-wide significance threshold (*p* < 3.846E-9), with 325 (87%, all located on chromosome 10) SNPs being associated with the S-warfarin/R-warfarin outcome (top SNP rs11188082, *CYP2C19* intron variant, *p* = 1.55E-17). Approximately 69% of these SNPs were in linkage disequilibrium (*r* ^2^ > 0.8) with CYP2C9*8 (*n* = 216) and rs12777823 (*n* = 8). Using a pharmacokinetic approach, we have shown that variants other than CYP2C9*2 and CYP2C9*3 are more important in sub-Saharan black-Africans, mainly due to the allele frequencies. In exploratory work, we conducted the first warfarin pharmacokinetics-related GWAS in sub-Saharan Africans and identified novel SNPs that will require external replication and functional characterization before they can be considered for inclusion in warfarin dosing algorithms.

## 1 Introduction

There is increasing acceptance of the need to increase diversity in genomic studies including in pharmacogenomics (Sirugo et al., 2019; Asiimwe and Pirmohamed, 2022; Fatumo et al., 2022). This is especially true in relation to the inclusion of black African patients, who have not been well-represented in previous studies (Sirugo et al., 2019; Asiimwe and Pirmohamed, 2022; Fatumo et al., 2022). Pharmacogenomic studies in sub-Saharan Africa have been hampered for several reasons: lack of critical mass of researchers in Africa interested in pharmacogenomics; lack of suitable funding; difficulties in recruiting patients because of the lack of infrastructure, which is especially important in pharmacogenomics, where there is often a need for deep phenotyping; and the huge genetic diversity in the African population which therefore requires large sample sizes (Tishkoff et al., 2009; Teo et al., 2010).

Due to low cost, warfarin remains the oral anticoagulant of choice in sub-Saharan Africa (Semakula et al., 2021). However, it has several dosing challenges including a narrow therapeutic window, and wide inter-patient dose-requirement variability due to genetic and non-genetic factors. These have contributed to the low anticoagulation quality in sub-Saharan Africa (Mouton et al., 2021), with warfarin being the leading cause of preventable adverse drug reaction-related hospitalizations in South Africa (Mouton et al., 2016).

To improve warfarin dosing, many algorithms that incorporate genetic and non-genetic factors are in use (Asiimwe et al., 2020b). Among the most commonly included genetic factors are the single nucleotide polymorphisms (SNPs) in the gene *CYP2C9* (cytochrome P450, family 2, subfamily C, polypeptide 9) that encodes the most important warfarin metabolizing enzyme (Asiimwe et al., 2020b). Due to minor allele frequencies, these SNPs (specifically rs1799853, CYP2C9*2 and rs1057910, CYP2C9*3) are more applicable to white and Asian populations as compared to black or other minority populations, in which other variants are believed to be more important (Cavallari and Perera, 2012). The United States Association for Molecular Pathology and College of American Pathologists in their joint recommendation for clinical *CYP2C9* genotyping allele selection include CYP2C9*2, CYP2C9*3, rs28371686 (CYP2C9*5), rs9332131 (CYP2C9*6), rs7900194 (CYP2C9*8), and rs28371685 (CYP2C9*11) as tier 1 variant alleles that should as a mimimum be included in clinical pharmacogenomic genotyping assays (Pratt et al., 2019). However, evidence for these alleles is mostly from studies conducted outside of sub-Saharan Africa (Asiimwe et al., 2019) and therefore more sub-Saharan African studies are required before these recommendations can be adopted in the region, and to fully characterize region-specific variants.

To identify and/or confirm genetic variants that are important in influencing warfarin dosing in patients of black-African ancestry, studies have utilised candidate gene and genome-wide approaches using stable warfarin dose as the outcome variable (Asiimwe et al., 2019). Alternatively, other studies (in other populations) have also used plasma concentrations of warfarin enantiomers and/or their metabolites to identify/confirm genetic variants of importance in warfarin pharmacokinetics, and consequently warfarin dosing (Scordo et al., 2002; Muszkat et al., 2007). This is important for several reasons: 1) it is the concentration of a drug/metabolite in the circulation or in tissue, rather than the dose per se, that determines its pharmacologic effect; 2) adherence patterns significantly impact study power (Mallayasamy et al., 2018), which means very large sample sizes will be required in sub-Saharan African studies with dose as an outcome, since more than half of patients in this population consider themselves non-adherent to warfarin dosing (Mouton et al., 2021); and 3) the genetic diversity of African populations (Tishkoff et al., 2009) further increases sample size requirements. Since recruiting participants from resource-limited settings can be very challenging (Teo et al., 2010), a pharmacokinetic approach that directly measures drug or metabolite concentrations, and circumvents adherence issues, has some advantages as it is likely to be the most powerful and sensitive in identifying genetic variants.

Warfarin is administered as a racemic mixture of the R (+) and S (−) stereoisomers [with the S-stereoisomer being 3–5 times more potent than its counterpart: half-maximal inhibitory concentration, IC50, for vitamin K epoxide reductase complex subunit 1 (VKORC1) being 288 and 25 nM for the R- and S-stereoisomers, respectively] (Choonara et al., 1986; Pirmohamed et al., 2015). Following oral administration, warfarin sodium is rapidly absorbed (79%–100% bioavailability) with considerable inter- and intra-individual variation in absorption rates. It is 99% protein-bound (and thus approximately 1% of the unbound drug is responsible for its pharmacologic effects) and has a relatively small apparent volume of distribution of 0.14 L/kg (Bristol-Myers Squibb, 2018; U.S. National Library of Medicine, 2018). Warfarin has a half-life of between 20 and 60 h (R-warfarin, 37–89 h; S-warfarin, 21–43 h) and is eliminated almost entirely by metabolism. Both stereoisomers undergo cytochrome P450 (CYP450)-mediated metabolism (key enzymes and metabolites shown in Supplementary Figure S1) with the major metabolites and enzymes differing between the two isomers (7- and 6-hydroxywarfarin from S-warfarin by CYP2C9 versus 10-hydroxywarfarin from R-warfarin by CYP3A4) (Shaik et al., 2016; Bristol-Myers Squibb, 2018; U.S. National Library of Medicine, 2018). Since *CYP2C9* is the main metabolizing enzyme for warfarin, its clearance mainly depends on *CYP2C9* genotype and in Whites, S-warfarin clearance has been estimated to be 0.065 ml/min/kg for CYP2C9*1*1 genotype, 0.041 ml/min/kg for *1*2/*1*3 genotypes, and 0.020 mg/min/kg for *2*2/*2*3/*3*3 genotypes (Bristol-Myers Squibb, 2018; U.S. National Library of Medicine, 2018).

The clearance of R-warfarin is approximately half of that of S-warfarin (Bristol-Myers Squibb, 2018; U.S. National Library of Medicine, 2018), resulting in an (S)-/(R)-warfarin ratio of approximately 0.5. A change in the activity of any of the metabolizing enzymes is likely to change this ratio and can be used as a basis of discovering new genetic variants involved in the pharmacokinetics of warfarin. In the present study, we have conducted the first genome-wide association study (GWAS) of plasma concentrations of warfarin enantiomers and metabolites in sub-Saharan black-African participants on stable warfarin dose in order to replicate previous findings and identify novel genetic variants of importance in any of the investigated warfarin pharmacokinetics-related outcomes, and consequently warfarin dosing in these populations.

## 2 Materials and methods

This study adheres to the STrengthening the Reporting Of Pharmacogenetic Studies (STROPS) guideline (Chaplin et al., 2020) (Supplementary Table S1).

### 2.1 Study design, setting and participants

This study included warfarin-treated adult (≥18 years) participants of self-reported black-African ethnicity who were recruited as part of an observational study by the National Institute of Health Research (NIHR) Global Health Research Group on WARfarin anticoagulation in PATients in Sub-Saharan Africa (War-PATH, http://warpath.info/; ClinicalTrials.gov Identifier: NCT03512080) as previously reported (Asiimwe et al., 2020a). Participants were recruited from 12 outpatient clinics and hospital departments in Uganda and South Africa between June 2018 and March 2020 (Asiimwe et al., 2020a; Asiimwe et al., 2021). The studies involving human participants were reviewed and approved by institutional review boards of the University of Liverpool (UK; ref: 2934), University of Cape Town (South Africa; ref: 672/2017), and Joint Clinical Research Centre (Uganda; ref: JC3017). Work in Uganda was also approved by the Uganda National Council for Science and Technology (ref: HS164ES). The patients/participants provided their written informed consent to participate in this study.

All included participants had achieved stable warfarin dose in the year preceeding recruitment, defined as the unchanged dose for two consecutive clinic visits, with the international normalized ratio (INR) being in the therapeutic range (2.0–3.0 for those with venous thromboembolism or atrial fibrillation and 2.5–3.5 for those with valvular heart disease) at both visits. Patients who were unwilling to take part, pregnant women or patients with any other contraindications based on clinician judgement were excluded as previously detailed (Asiimwe et al., 2020a). Participants whose blood samples were not available at the time of DNA analysis were also excluded.

### 2.2 Variables

The study end points were: the individual and combined concentrations of the warfarin enantiomers (S-warfarin, R-warfarin, RS-warfarin), the main metabolites (S-6OH-warfarin, R-6OH-warfarin, RS-6OH-warfarin, S-7OH-warfarin, RS-10-hydroxywarfarin) and analyte ratios (S-warfarin/R-warfarin, S-6OH-warfarin/S-warfarin, R-6OH-warfarin/R-warfarin, S-7OH-warfarin/S-warfarin, RS-10hydroxywarfarin/RS-warfarin). R-4OH-warfarin and R-7OH-warfarin were not included as they were quantified in fewer than 100 participants, while enantiomers for 10-hydroxy-warfarin were not determined separately (see the “*Data sources/measurement*” section).

To replicate previous reports (Asiimwe et al., 2019; Pratt et al., 2019), we first considered the SNPs that are known to be important in warfarin pharmacokinetics (CYP2C9*6, CYP2C9*8, CYP2C9*11, and rs12777823) as exposures. Although the rs2256871 (CYP2C9*9) missense variant (histidine to arginine change at position 251) has minimal effect on enzyme function (Pratt et al., 2019), slightly decreased activity towards S-warfarin compared with the wild-type has been reported for the protein encoded by this variant (Niinuma et al., 2014) and so it was also considered an additional exposure. We could not include the SNPs rs1799853 (CYP2C9*2), rs1057910 (CYP2C9*3) and rs28371686 (CYP2C9*5) in this analysis as they failed the imputation/r-squared and minor allele frequency (MAF) thresholds during genotype quality control (details under “*Data sources/measurement*” and “*Results*” sections). The widely known *VKORC1* (warfarin’s molecular target) and *CYP4F2* (a vitamin K oxidase) SNPs (rs7294*, VKORC1 3730G>A*; rs2359612, *VKORC1 2255C>T*; rs8050894, *VKORC1 1542G>C*; rs9934438, *VKORC1 1173C>T*; rs2884737, *VKORC1 497T>G*; rs9923231, *VKORC1 −1639G>A*; rs2108622, CYP4F2*3) were also included as “negative” controls since we don’t expect them to affect warfarin pharmacokinetics. All other genotyped and imputed SNPs (details under “*Data sources/measurement*”) that passed genotype quality control were considered as exposures in an exploratory GWAS analysis.

Five non-genetic covariates (age, sex, weight, simvastatin/amiodarone status, and efavirenz status) and ten principal components of genetic ancestry were considered as additional predictor variables during analysis. Four of the above non-genetic variables (age, sex, weight, and simvastatin/amiodarone status) were previously selected based on expert guidance and literature review during the development of the War-PATH clinical dose-initiation algorithm (Asiimwe et al., 2020a). Three other previously selected non-genetic variables [country of recruitment, target INR range, and human immunodeficiency virus (HIV) status] were not considered for the following reasons. First, country of recruitment was included as a proxy for underlying population substructure; with GWAS data available in this analysis, the ten principal components of genetic ancestry (Price et al., 2006) were preferable as they are more accurate. Second, a higher target INR range implies a stronger pharmacodynamic effect is required, which does not support the adjustment of this variable in pharmacokinetic analysis. Lastly, HIV infection affects warfarin response through pharmacodynamics (e.g., leading to a hypercoaguable state) or pharmacokinetics (interactions with antiretroviral drugs) (Asiimwe et al., 2020a). In this pharmacokinetic study, exploring efavirez status [efavirenz is predicted to affect warfarin’s concentration; moderate severity (Joint Formulary Committee, 2019)] as a covariate was therefore more suitable than HIV status. Other antiretrovirals can affect warfarin concentrations (Liedtke and Rathbun, 2009), however, they were not considered as covariates as they were not taken or were taken by very few patients.

### 2.3 Data sources/measurement

#### 2.3.1 Warfarin and metabolite concentrations

After K3-EDTA plasma samples were acquired during patient enrolment, a chiral high-performance liquid chromatography-tandem mass spectrometry (HPLC-MS/MS) assay was developed and validated to quantify warfarin and its five major metabolites (4′-, 6-, 7-, 8-, and 10-OH warfarin). The assay was optimized to detect R-warfarin, S-warfarin, R-4-OH-warfarin, S-4-OH-warfarin, R-6-OH-warfarin, S-6-OH-warfarin, R-7-OH-warfarin, S-7-OH-warfarin, R-8-OH-warfarin, S-8-OH-warfarin, and racemic 10-OH warfarin (Supplementary Figure S2). Full validation criteria were met for calibration curve performance, carryover, selectivity, dilution integrity, matrix effect, extraction recovery, accuracy, precision, plasma stability, and autosampler stability. Results of the bioanalytical method validation are summarised in Supplementary Table S2. The within run and between run accuracy of the lower limit of quantification (LLOQ), low, medium and high quality controls for all analytes were within the accepted range, precision coefficient of variation (CV) was 1.3%–12.7%. After 4-fold dilution with blank plasma, the accuracy and precision CV of all analytes was 94.7%–107.0% and 0.7%–5.0% respectively; as such patient samples higher than the upper limit of quantification (ULOQ) can be diluted up to four times. All analytes were stable in plasma on the benchtop (9 h, room temperature), in the fridge (24 h, 4–8°C), after five freeze-thaw cycles (storage at approximately −80°C), and in the autosampler (30 h, set at 4°C). Other details (chromatographic solvents and buffers, sample preparation and data acquisition methods) are shown in Supplementary Text S1 and Supplementary Table S3.

Raw chromatographic data were processed using the Analyst® 1.6.2 and MultiQuant™ version 3.0 (AB Sciex, UK) softwares. Accuracy ranges were set as ±20% for the lowest concentration (LLOQ) and ±15% for all other concentrations in the calibration curve, including the top calibration standard (ULOQ). All analytical runs had a minimum of seven calibration standards, with over 88% of the calibration standards fulfilling the above-mentioned accuracy range. A minimum of three quality control concentration levels (low, medium, high) were included per run with all calibration standards and quality controls run in duplicate. The dynamic range for the parent compounds was 200–8,000 ng/ml. For 10-OH-warfarin, the dynamic range was 10 times lower, 20–800 ng/ml. All other metabolites had LLOQ and ULOQ of 25 ng/ml and 1,000 ng/ml respectively. Samples with values below the LLOQ or above the ULOQ were excluded from analysis.

#### 2.3.2 Clinical data

Data pertaining to age, sex, weight, simvastatin/amiodarone and efavirenz prescription were captured using War-PATH study case report forms during enrolment as detailed in our previous report (Asiimwe et al., 2020a).

#### 2.3.3 DNA extraction and genotyping

During enrolment, 5 ml whole blood was drawn in K3-EDTA vacutainers from each participant. To inactivate any viable *Mycobacterium tuberculosis* and/or human immunodeficiency virus present in the blood samples, chemical and heat inactivation was carried out using 1.5 volumes of 2X lysis buffer (PerkinElmer chemagen Technologie GmbH, UK) at 58°C for 45 min in a biosafety level 3 laboratory prior to DNA extraction (University of Liverpool, United Kingdom). DNA was subsequently extracted from the inactivated blood samples using the chemagic Magnetic Separation Module (MSM) I instrument (PerkinElmer chemagen Technologie GmbH, UK) according to the manufacturer’s guidelines in a biosafety level 2 laboratory (University of Liverpool, United Kingdom). Extracted DNA samples that showed good quality with A260/A280 ratios between 1.8 and 2.0 were normalised (50 ng/µl) and shipped to the Cambridge Genomics Services, a genotyping service provider at the University of Cambridge (Cambridge, United Kingdom). A minimum of 2 μg DNA was used for genome-wide genotyping on the Illumina Infinium H3Africa Consortium Array v2 containing 2,271,503 SNPs according to the manufacturer’s instructions (Illumina, San Diego, CA, United States). Genotyping personnel were not aware of the outcome status of included patients. To ensure quality assurance, at least five duplicates were included in each 96-well plate.

#### 2.3.4 Genotyping quality control and imputation

Prior to analyses of association, both per patient and per SNP quality control criteria were applied to the genotype data. Patients were excluded from analysis if they failed to meet the following criteria:

a) Sex, determined by the “Sex Check” function within PLINK (Purcell et al., 2007), being consistent with clinical information;
b) Genotype call-rate ≥ 95%;
c) Principal component analysis (PCA) demonstrating that the participants clustered with the 1,000 genomes African populations (Genomes Project et al., 2010);
d) Were unrelated to other participants (based on an identity-by-descent coefficient cut-off of 0.1875 in a pruned subset of uncorrelated SNPs) or if they were related to another participant, had a lower amount of missingness than them; or,
e) Non-extreme heterozygosity (identified from a plot of mean heterozygosity versus proportion missing genotypes).

On the other hand, SNPs were excluded if:

a) The minor allele frequency (MAF) was <0.01;
b) Hardy-Weinberg Equilibrium *P* value was <0.000001; or,
c) The genotype success rate was <95%.

Unless otherwise stated, all QC analysis (details of commands used in Supplementary Text S2) were conducted using PLINK v1.9 (Purcell et al., 2007). After the above QC steps, the accuracy relative to the 1,000 Genomes phase III reference panel (Genomes Project et al., 2010) was checked to minimize imputation errors, following the steps available on the Michigan imputation server (https://imputationserver.sph.umich.edu/index.html#!) (Das et al., 2016). Genotype imputation was conducted using the same server, with pre-phasing and imputation being conducted using SHAPEIT v2 (Delaneau et al., 2011) and IMPUTE2 (Howie et al., 2009) software, respectively. Finally, post-imputation QC involved filtering out SNPs with low imputation accuracies (those with an r-squared (Browning and Browning, 2009) < 0.3 excluded) and low MAF (those with MAF <0.01 excluded).

### 2.4 Study size calculation

No formal sample size calculations were conducted. However, all eligible participants were included in the analysis to maximize the replication/discovery sample sizes, with a minimum sample size of 100 participants (default internal limit of SNPTEST Version 2 (Marchini and Howie, 2010) which was used for analysis).

### 2.5 Statistical methods

#### 2.5.1 Outcome transformation

We logarithmically transformed the outcomes to achieve a normal distribution as well as to obtain a proportional/multiplicative scale that is easy to interpret (Keene, 1995; Vittinghoff et al., 2012).

#### 2.5.2 Handling quantitative predictors

Quantitative predictor variables were neither transformed nor categorized.

#### 2.5.3 Missing data

Missing genotype data was imputed using the Michigan imputation server (Das et al., 2016) as described above, using IMPUTE2 (Howie et al., 2009). For consistency with previous work (Asiimwe et al., 2020a), missing weight information (*n* = 11 cases, 2%) was imputed using single imputation (predictive mean matching) within the Multivariate Imputation by Chained Equations (MICE) R package (van Buuren and Groothuis-Oudshoorn, 2011) [based on non-genetic covariates (including age, weight, sex, target INR, HIV status, simvastatin/amiodarone status), additional genetic covariates (ten principal components of genetic ancestry) and outcome (weekly stable warfarin dose)]. We included weekly stable warfarin dose in the imputation model since including outcomes such as stable warfarin dose is preferred over their non-inclusion (Moons et al., 2006).

#### 2.5.4 Analysis

Following genotype QC, a multivariable linear regression model was fitted with each SNP in turn as a predictor variable assuming an additive mode of inheritance, and each of the above mentioned study endpoints (warfarin concentrations, metabolite concentrations and analyte ratios) as the outcomes. We adjusted for ten principal components of genetic ancestry as well as age, sex, weight, and intake of simvastatin, amiodarone and/or efavirenz. For the replication analysis of known variants, we used a Bonferroni-adjusted replication significance threshold (0.05 divided by 12 SNPs divided by 13 outcomes = *p* < 3.21 × 10^−4^).

We used two statistical significance thresholds for the exploratory GWAS analysis: a Bonferroni multiple testing-corrected genome-wide statistical significance threshold of *p* < 5 × 10^−8^/13 outcomes = *p* < 3.846 × 10^−9^ (to reduce false positives), as well as a nominal significance threshold of *p* < 1 × 10^−5^. We conducted two sensitivity analyses: a complete case analysis in which the 11 cases missing weight were excluded from analysis (to check the accuracy of the single-imputation approach for weight), and a model-based analysis (to check if multi-marker models would detect signals that single marker analysis fails to detect in complex traits) (Frommlet et al., 2012; Dolejsi et al., 2014; Hofer et al., 2017). Model-based linear regression analysis was implemented using the software package MOSGWA (Model Selection for Genom-Wide Associations, http://mosgwa.sourceforge.net), which is based on a modification of the Bayesian Information Criterion (Dolejsi et al., 2014), using default parameters. Other analyses were undertaken using SNPTEST Version 2 (Marchini and Howie, 2010) using threshold genotypes (default calling threshold of 0.9) and the results graphically presented using Manhattan and Quantile-Quantile plots [qqman R package (Turner, 2018)]. The Single Nucleotide Polymorphism Database (dbSNP, https://www.ncbi.nlm.nih.gov/snp/) (Smigielski et al., 2000; Kitts and Sherry, 2011) was used to obtain the gene and location/functional consequences of SNPs passing the nominal significance threshold, while the Genotype-Tissue-Expression (GTEx) analysis release V8 (https://www.gtexportal.org/home/) (Consortium, 2013) was used to obtain expression quantitative trait loci (eQTL) and splicing quantitative trait loci (sQTL) for SNPs passing the Bonferroni-adjusted genome-wide statistical significance threshold. Regions of potential genomic interest were further investigated using regional locus plots (LocusZoom v0.4.8, http://locuszoom.org/) (Pruim et al., 2010) while Haploview (v4.2) (Barrett et al., 2005) was used for linkage disequilibrium analysis (*r* ^2^ threshold = 0.8). Key statistical analysis codes/commands are available in Supplementary Text S2.

## 3 Results

### 3.1 Participants

**FIGURE 1:**
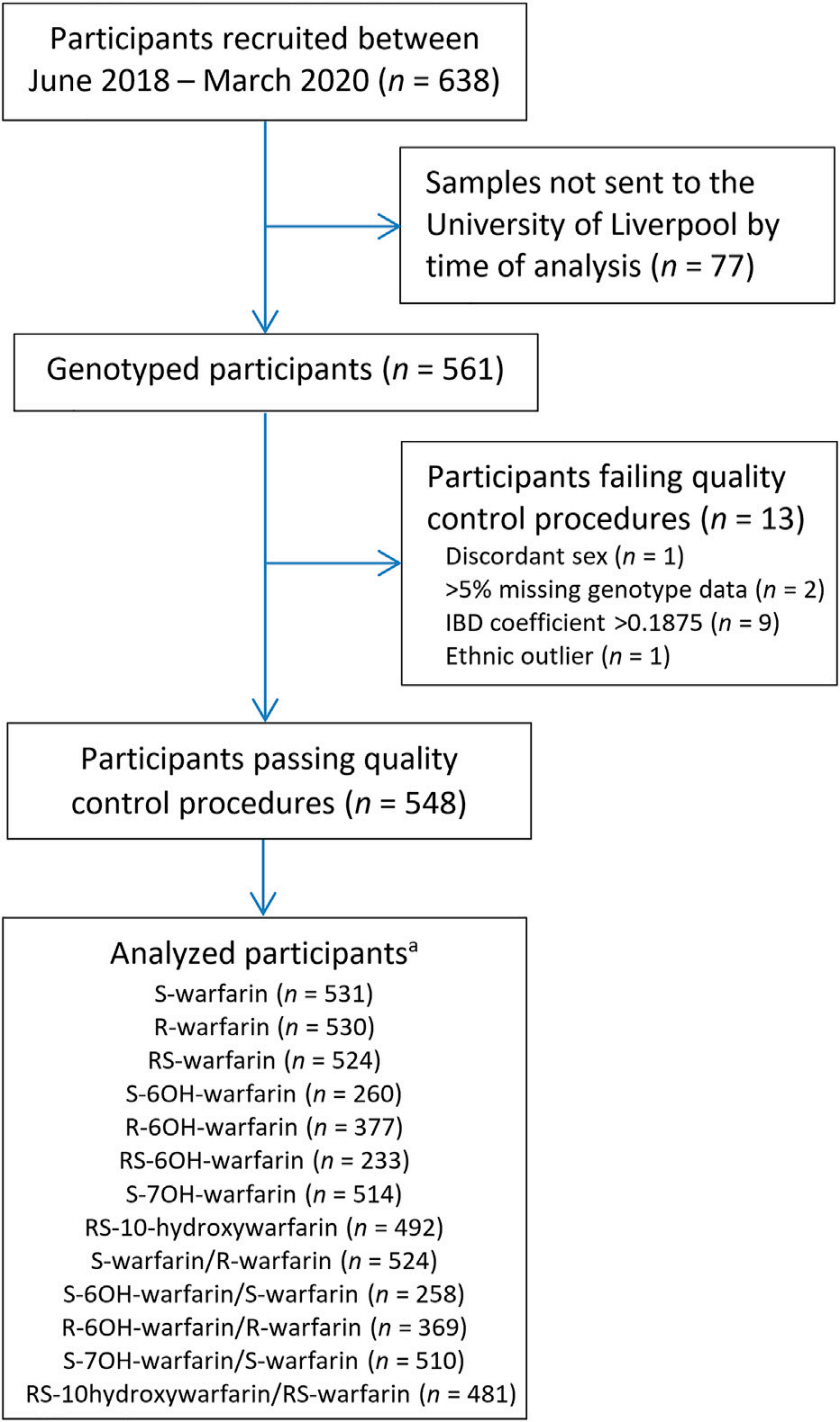
Flow chart for included participants. ^a^Only patients who were within the dynamic range included in analysis. IBD = identity-by-descent.

Out of 638 eligible black-African participants recruited from Uganda and South Africa between June 2018 and March 2020, 561 samples had been shipped to the University of Liverpool by the time of analysis. During per-sample/per-individual quality control (QC), one individual was excluded due to discordant clinical/X-chromosome-derived sex, two individuals due to having genotype call rates <95%, one due to being an ethnic outlier and nine due to having an identity-by-descent coefficient greater than 0.1875 (Figure 1; Supplementary Figure S3) leaving a total of 548 individuals, whose characteristics are shown in Table 1. Only the participants with warfarin/metabolite concentrations within the dynamic range were included in the corresponding analyses, with the sample sizes ranging from 233 (RS-6OH-warfarin endpoint) to 531 (S-warfarin endpoint) as shown in Figure 1 and Table 1. Supplementary Figure S4 shows the correlations between the study end-points while Supplementary Figure S5 shows quantile–quantile plots before and after applying a logarithmic transformation to the study end-points. A comparison of participants who were included versus those excluded from a particular analysis is shown in Supplementary Table S4.

**TABLE 1:** Clinical/demographic characteristics of the participants who passed quality control procedures (*N* = 548).

Variables	Frequency (%) or median (IQR)
Country of recruitment	
South Africa	287 (52.37%)
Uganda	261 (47.63%)
Age (years)	45.62 (34.78–57.00)
Sex	
Female	390 (71.17%)
Male	158 (28.83%)
Weight (kg, *n* = 537)	72.00 (60.00–87.00)
INR Target range	
2.0–3.0	357 (65.15%)
2.5–3.5	191 (34.85%)
HIV status	
Negative	428 (78.10%)
Positive	97 (17.70%)
Unknown	23 (4.20%)
Efavirenz	
Yes	65 (11.86%)
No	483 (88.14%)
Simvastatin/Amiodarone	
Yes	51 (9.31%)
No	497 (90.69%)
Weekly stable dose (mg)	35.00 (30.00–50.00)
Analyte concentrations (ng/ml)	
S-warfarin (*n* = 531)	1811.17 (1,327.28–2,367.56)
R-warfarin (*n* = 530)	3,160.27 (2,304.51–4,199.45)
RS-warfarin (*n* = 524)	5,073.16 (3,825.40–6,316.03)
S-6OH-warfarin (*n* = 260)	37.03 (30.46–49.62)
R-6OH-warfarin (*n* = 377)	60.21 (40.37–101.77)
RS-6OH-warfarin (*n* = 233)	118.62 (85.93–175.74)
S-7OH-warfarin (*n* = 514)	274.23 (160.70–444.31)
RS-10-hydroxywarfarin (n = 492)	64.55 (42.79–106.12)
Analyte concentration ratios	
S-warfarin/R-warfarin (*n* = 524)	0.56 (0.43–0.73)
S-6OH-warfarin/S-warfarin (*n* = 258)	0.02 (0.01–0.03)
R-6OH-warfarin/R-warfarin (*n* = 369)	0.02 (0.01–0.03)
S-7OH-warfarin/S-warfarin (*n* = 510)	0.15 (0.09–0.26)
RS-10hydroxywarfarin/RS-warfarin (*n* = 481)	0.01 (0.01–0.02)

HIV, human immunodeficiency virus; INR, international normalized ratio; IQR, interquartile range.

### 3.2 Single nucleotide polymorphisms

A total of 2,271,503 SNPs were genotyped using the Illumina Infinium H3Africa Consortium Array v2. Per-SNP QC excluded 344,443 SNPs based on genotype success rate <95% (*n* = 41,460), Hardy-Weinberg Equilibrium *p* value < 0.000001 (*n* = 4,215), and minor allele frequency <0.01 (*n* = 298,768) resulting in a total of 1,927,060 SNPs remaining. After more SNPs were added during the imputation process (and after applying post-imputation QC steps), a total of 17,268,054 SNPs were included in the final analysis.

### 3.3 Single nucleotide polymorphisms known to influence warfarin pharmacokinetics

Table 2 shows the three outcomes (S-warfarin/R-warfarin, S-6OH-warfarin/S-warfarin, S-7OH-warfarin/S-warfarin) for which we were able to replicate previous reports. Specifically, CYP2C9*8 (S-warfarin/R-warfarin *p* = 9.53 × 10^−12^, S-7OH-warfarin/S-warfarin *p* = 6.68 × 10^−5^), CYP2C9*9 (S-warfarin/R-warfarin *p* = 8.54 × 10^−8^, S-6OH-warfarin/S-warfarin *p* = 1.70 × 10^−4^), and CYP2C9*11 (S-warfarin/R-warfarin *p* = 9.00 × 10^−9^, S-7OH-warfarin/S-warfarin *p* = 1.00 × 10^−5^) were shown to significantly influence warfarin pharmacokinetics after adjustment for multiple testing (Bonferroni-adjusted replication significance threshold *p* < 3.21 × 10^−4^). The *CYP2C* cluster SNP rs12777823 was also replicated based on the outcomes S-warfarin/R-warfarin (*p* = 3.61 × 10^−8^) and S-7OH-warfarin/S-warfarin (*p* = 5.07 × 10^−7^). As expected, none of the negative controls (using the known *VKORC1* and *CYP4F2* SNPs) were statistically significant. The other ten outcomes that did not have any statistically significant results are shown in Supplementary Table S5.

**TABLE 2:** *P*-values for widely-known SNPs^a^.

#	rsID (Reference/alternative alleles)	Common name	S-warfarin/R-warfarin (*N* = 524)			S-6OH-warfarin/S-warfarin (*N* = 258)			S-7OH-warfarin/S-warfarin (*N* = 510)		
			MAF	Beta^b^ (SE)	*p*-value	MAF	Beta^b^ (SE)	*p*-value	MAF	Beta^b^ (SE)	*p*-value
1	rs12777823 (G/A)	NA	0.279	0.330 (0.059)	*3.61E-08*	0.248	−0.257 (0.103)	1.31E-02	0.278	−0.314 (0.062)	*5.07E-07*
2	rs9332131 (GA/G)	CYP2C9*6	0.010	−0.320 (0.270)	2.37E-01	0.012	0.223 (0.408)	5.85E-01	0.010	−0.160 (0.290)	5.80E-01
3	rs7900194 (G/A)	CYP2C9*8	0.071	0.698 (0.100)	*9.53E-12*	0.054	−0.267 (0.191)	1.62E-01	0.072	−0.427 (0.106)	*6.68E-05*
4	rs2256871 (A/G)	CYP2C9*9	0.168	0.372 (0.068)	*8.54E-08*	0.159	−0.454 (0.119)	*1.70E-04*	0.172	−0.205 (0.072)	4.49E-03
5	rs28371685 (C/T)	CYP2C9*11	0.022	1.063 (0.182)	*9.00E-09*	0.010	−1.436 (0.429)	9.47E-04	0.022	−0.861 (0.193)	*1.00E-05*
6	rs7294 (G/A)	*VKORC1 3730G>A*	0.483	0.028 (0.058)	6.32E-01	0.440	−0.111 (0.090)	2.20E-01	0.484	−0.055 (0.060)	3.61E-01
7	rs2359612 (C/T)	*VKORC1 2255C>T*	0.222	−0.075 (0.066)	2.59E-01	0.238	−0.136 (0.100)	1.77E-01	0.223	−0.047 (0.068)	4.89E-01
8	rs8050894 (G/C)	*VKORC1 1542G>C*	0.204	−0.148 (0.067)	2.88E-02	0.194	0.304 (0.112)	6.87E-03	0.202	0.078 (0.071)	2.74E-01
9	rs9934438 (C/T)	*VKORC1 1173C>T*	0.053	−0.104 (0.125)	4.05E-01	0.048	0.656 (0.203)	1.39E-03	0.052	0.215 (0.131)	1.01E-01
10	rs2884737 (T/G)	*VKORC1 497T>G*	0.019	−0.307 (0.200)	1.26E-01	0.019	0.527 (0.312)	9.21E-02	0.018	0.321 (0.215)	1.35E-01
11	rs9923231 (G/A)	*VKORC1 -1639G>A*	0.052	−0.105 (0.126)	4.04E-01	0.047	0.679 (0.207)	1.19E-03	0.051	0.218 (0.132)	9.92E-02
12	rs2108622 (C/T)	CYP4F2*3	0.063	0.022 (0.116)	8.50E-01	0.058	−0.393 (0.191)	4.10E-02	0.066	−0.030 (0.119)	8.02E-01

a The SNP rs1799853 (CYP2C9*2) was not in the Illumina Infinium H3Africa Consortium Array v2 panel and could not be successfully imputed (*R* ^2^ = 22.3%), while SNPs rs1057910 (CYP2C9*3) and rs28371686 (CYP2C9*5) did not pass the MAF threshold post imputation (respective MAFs 0.009 and 0.007 in the 548 participants passing quality control procedures).

b Coefficient of the alternative allele relative to the reference allele. Abbreviations: MAF, minor allele frequency; NA, not applicable; OH, hydroxyl; rsID, reference; SNP, cluster ID; SE, standard error; SNP, single nucleotide polymorphism.

Gene names and *p*-values passing the Bonferroni-adjusted replication significance threshold *p* < 3.21 × 10^−4^ are italicized.

### 3.4 Exploratory genome-wide association analysis

**FIGURE 2:**
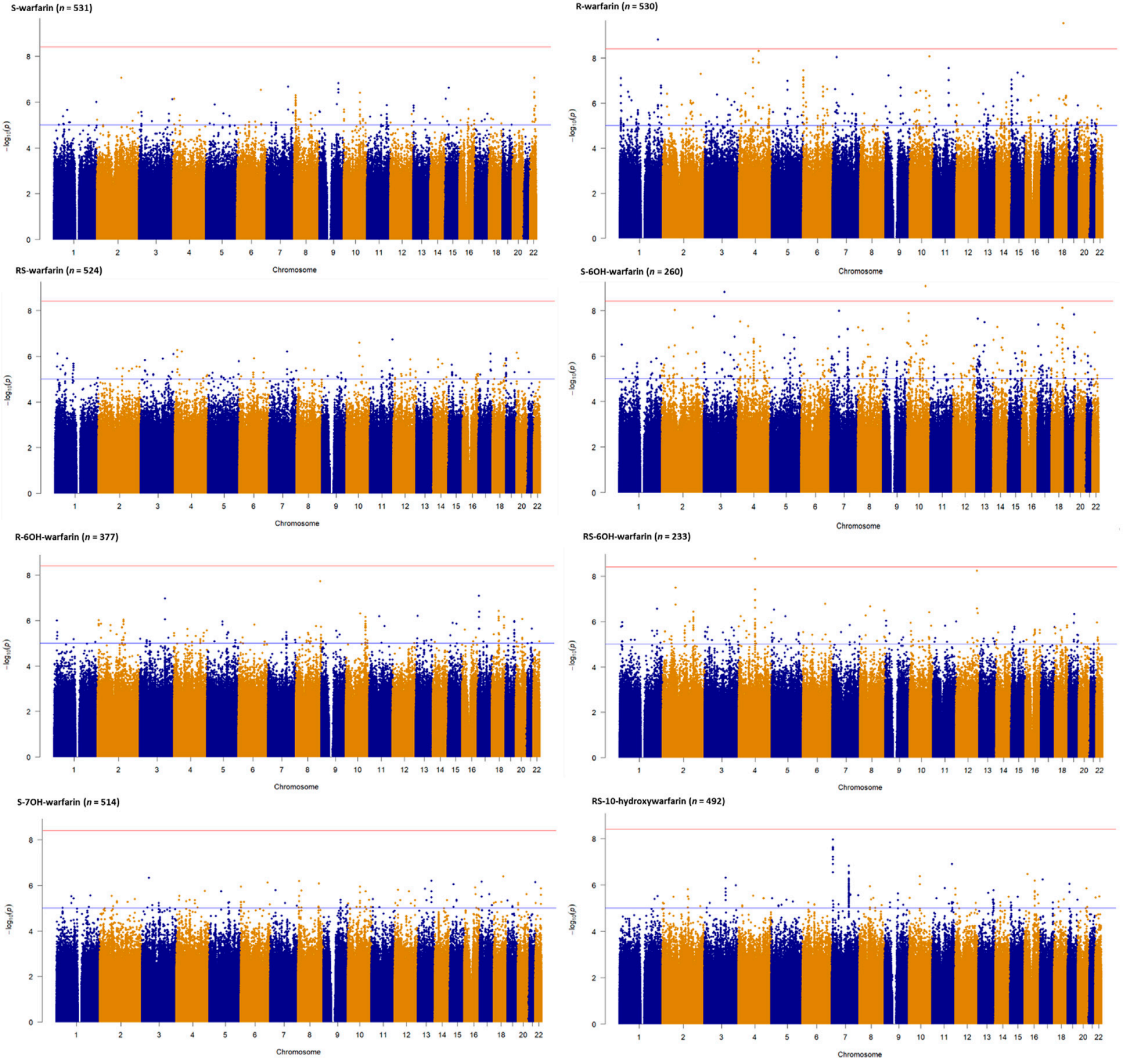
Manhattan plots of warfarin enantiomers and metabolites. Genome-wide association analyses were carried out using natural logarithm transformed analyte concentrations, adjusted for age, sex, weight, simvastatin/amiodarone and efavirenz statuses, and ten principal components by frequentist association testing assuming an additive model of inheritance.

**FIGURE 3:**
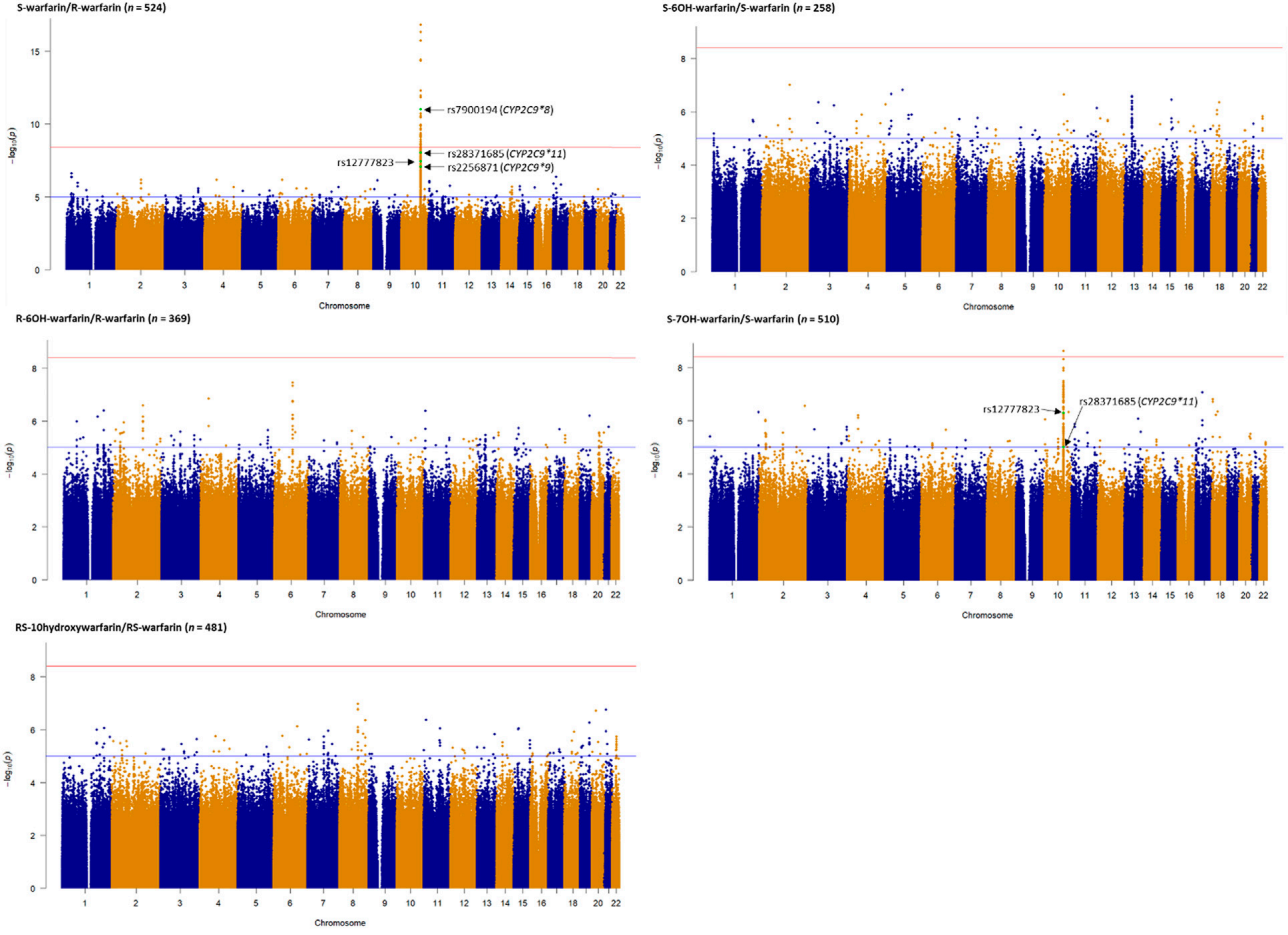
Manhattan plots of analyte (warfarin enantiomers, metabolites) ratios. Genome-wide association analyses were carried out using natural logarithm transformed analyte concentration ratios, adjusted for age, sex, weight, simvastatin/amiodarone status and efavirenz statuses, and ten principal components by frequentist association testing assuming an additive model of inheritance. The top SNPs already known to significantly influence warfarin pharmacokinetics are annotated.

Figure 2 shows the Manhattan plots for the individual and combined concentrations of the warfarin enantiomers (S-warfarin, R-warfarin, RS-warfarin) as well as the key metabolites (S-6OH-warfarin, R-6OH-warfarin, RS-6OH-warfarin, S-7OH-warfarin, RS-10-hydroxywarfarin) while Figure 3 shows the Manhattan plots for the analyte ratios (S-warfarin/R-warfarin, S-6OH-warfarin/S-warfarin, R-6OH-warfarin/R-warfarin, S-7OH-warfarin/S-warfarin, RS-10hydroxywarfarin/RS-warfarin). The corresponding quantile-quantile (QQ) plots showing limited evidence of genomic inflation are shown in Supplementary Figure S6.

**FIGURE 4:**
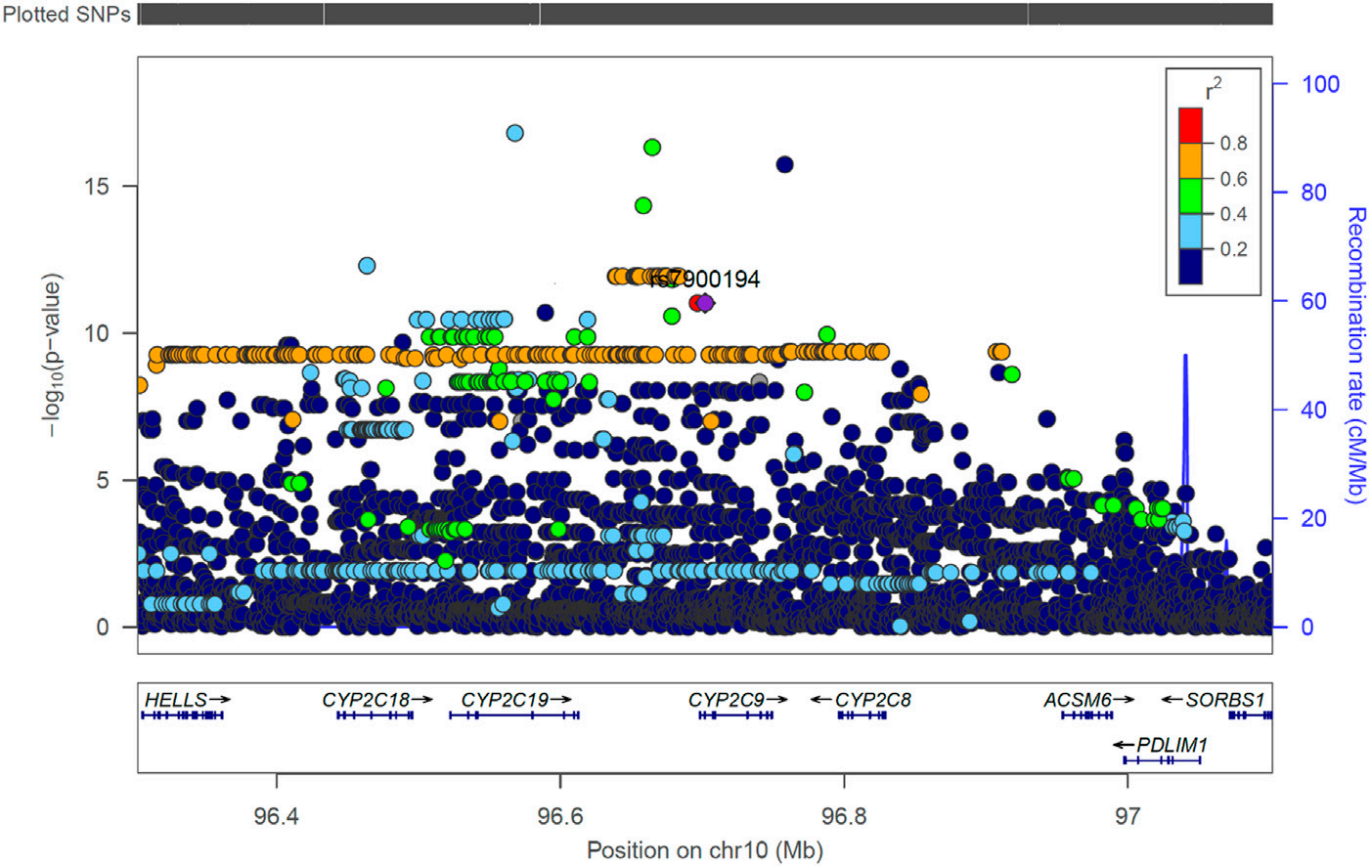
Regional LocusZoom plot of the established *CYP2C9* SNP rs7900194 (CYP2C9*8). The linkage disequilibrium (LD) pattern is based on the 1,000 genomes African populations (Genomes Project et al., 2010). In this study, most of the SNPs shown above to have an *r* ^2^ between 0.6 and 0.8 (orange circles) were in LD with rs7900194 (*r* ^2^ > 0.8).

Supplementary Tables S6–S18 show the SNPs with *p*-values lower than the nominal significance threshold (*p* < 1 × 10^−5^, 4,970 SNPs for all outcomes, with 4,524 SNPs being unique as some SNPs appeared in more than one outcome). Of these, 373 unique SNPs in 13 genes were genome-wide significant hits (*p* < 3.846 × 10^−9^, one SNP appeared in two outcomes), as detailed below:

• Two SNPs in two genes (rs115773951, *p* = 2.90 × 10^−10^, intron variant of myosin Vb [*MYO5B*] gene, chromosome 18; and, rs79414888, *p* = 1.58 × 10^−9^, intron variant of Rab3 GTPase activating non-catalytic protein subunit 2 [*RAB3GAP2*] gene, chromosome 1) were associated with the plasma concentrations of R-warfarin (*n* = 530).
• For S-6OH-warfarin (*n* = 260), there were six genome-wide significant hits (five [rs368245720, rs541817388, 10:107692518:AC:A, rs112552343, rs372488899] SNPs in the same intergenic region on chromosome 10 with *p* = 8.68 × 10^−10^; and rs10433340, *p* = 1.53 × 10^−9^, intron variant of poly(ADP-ribose) polymerase family member 14 [*PARP14*] gene, chromosome 3).
• For RS-6OH-warfarin (n = 233), there were 40 genome-wide significant hits lying in the same genomic region (all *p* = 1.72 × 10^−9^, intron variants of glutamate ionotropic receptor delta type subunit 2 [*GRID2*] gene).
• The S-warfarin/R-warfarin outcome (*n* = 524) had 325 genome-wide significant SNPs (lead SNP rs11188082, *p* = 1.55 × 10^−17^, intron variant of Cytochrome P450 [CYP] family 2 subfamily C member 19 [*CYP2C19*] gene). All these SNPs were located on chromosome 10 and found within/close to nine genes, of which four are included in the warfarin pharmacokinetic pathway (namely *CYP2C8*, *CYP2C9*, *CYP2C18*, and *CYP2C19*, Supplementary Figure S1). The well-established CYP2C9*8 missense variant was among the genome-wide significant hits (*p* = 9.53 × 10^−12^) and its regional plot, which also shows the above four genes is shown in Figure 4. Of the remaining 324 SNPs, 216 (66.7%) were in linkage disequilibrium (LD) with this CYP2C9*8 variant and these are shown in Supplementary Table S19, that also includes the eight SNPs in LD with rs12777823. As shown in Supplementary Table S14, most of the genome-wide significant hits were either intergenic or intronic. In addition to the above CYP2C9*8 missense variant, two other SNPs that require pointing out are rs9332241 (*p* = 5.38 × 10^−10^, three prime untranslated region variant of *CYP2C9*, in LD with CYP2C9*8) and rs41291550 (*p* = 3.70 × 10^−9^, stop gained coding sequence variant of *CYP2C18*).
• Lastly, the S-7OH-warfarin/S-warfarin outcome (*n* = 510) had one genome-wide significant hit (rs58800757, *p* = 2.33 × 10^−9^, upstream variant of uncharacterized *LOC107984256* gene, chromosome 10). This SNP also appeared in the S-warfarin/R-warfarin outcome.

Seventy-nine (21.2%) of the 373 unique genome-wide significant SNPs had statistically significant expression quantitative trait loci (eQTLs, Supplementary Table S20), including eight SNPs (rs7085563, rs12570829, rs34582766, rs12775423, rs12782132, rs7085420, rs1926711, rs35835168) that are significantly associated with the hepatic expression of *CYP2C19* (*p*-values ranging from 3.40 × 10^−6^ to 4.50 × 10^−5^). On the other hand, 75 (20.1%) of the 373 unique genome-wide significant SNPs had statistically significant splicing quantitative trait loci (sQTLs, Supplementary Table S21), including 30, 8, 8, and 4 SNPs significantly associated with the hepatic splicing of *CYP2C8*, *CYP2C18*, *CYP2C19*, and *CYP2C9* mRNAs, respectively (all *p*-values < 4.70 × 10^−7^).

Consistent with a slightly smaller sample size, most SNPs had less precise estimates (larger standard errors) in the complete-case analysis that was conducted in order to determine the robustness of the single imputation approach used to infer missing weight for 11 cases. This resulted in slightly less genome-wide significant SNPs (*n* = 362 unique SNPs, Supplementary Table S22). Lastly, in the model-based analysis, nine SNPs that had all been detected by single-marker analysis (Supplementary Tables S9, S11, S14, S17) were included in the final models for four of the outcomes, namely: S-6OH-warfarin (4 SNPs), RS-6OH-warfarin (1 SNP), S-warfarin/R-warfarin (3 SNPs) and S-7OH-warfarin/S-warfarin (1 SNP) as shown in Supplementary Table S23.

## 4 Discussion

In this study, we have undertaken a GWAS using plasma concentrations and ratios of warfarin and its metabolites in a sub-Saharan black African population to identify genetic determinants of warfarin pharmacokinetics, and thereby dose and response. Candidate gene studies and GWAS of plasma concentrations have been undertaken previously for a number of other drugs including acetaminophen, atorvastatin, bisoprolol, antidepressants and antipsychotics (Turner et al., 2020; Jukic et al., 2021; Milosavljevic et al., 2021; Fontana et al., 2022; Thareja et al., 2022). This has many advantages, as outlined in the introduction, but has disadvantages as well, including the fact that the approach increases the complexity and cost of studies, there is a need to set up validated drug/metabolite concentration analytical techniques and because of the rarity of such studies, it is more difficult to identify independent cohorts for replication.

Our report is the first warfarin pharmacokinetics-related genome-wide association study (GWAS) in sub-Saharan African individuals. We have undertaken a two-step analysis: a replication analysis, focusing on *CYP2C9* variants and the *CYP2C* gene cluster allele rs12777823 which are well known determinants of warfarin dose requirements, and an exploratory GWAS analysis. *CYP2C9* is the most important gene involved in the metabolism of S-warfarin, the more potent enantiomer. Reduced/null function variants (such as *2, *3, *5, *6, *8, and *11) (Rettie et al., 1994; Haining et al., 1996; Dickmann et al., 2001; Kidd et al., 2001; Allabi et al., 2004; Tai et al., 2005; Liu et al., 2012; Niinuma et al., 2014; Wanounou et al., 2022) lead to reduced S-warfarin metabolism, which increases the S-warfarin/R-warfarin ratio (positive beta coefficients, Table 2), as observed for CYP2C9*8 (*p* = 9.53 × 10^−12^) and CYP2C9*11 (*p* = 9.00 × 10^−9^). Consistent with the above results, the S-7OH-warfarin/S-warfarin ratio for these two variants decreased (negative beta coefficients (Table 2) are consistent with decreased metabolite and increased substrate amounts) with both results passing the Bonferroni-adjusted replication significance threshold (*p* < 3.21 × 10^−4^). The CYP2C9*2 variant was neither genotyped nor successfully imputed (*R* ^2^ = 22.3%) and so could not be included in analysis. Based on the 1,000 genomes African MAF of 0.8% for this variant (Genomes Project et al., 2010), it is likely that it would still have been excluded from analysis had it been genotyped/successfully imputed due to failing post imputation quality control checks (MAF <1%). Indeed, the latter was the reason for excluding CYP2C9*3 (MAF in the 548 patients passing quality control procedures = 0.9%) and CYP2C9*5 (MAF = 0.7%) variants. The very low MAF (1%) for CYP2C9*6 could explain its non-significant associations (e.g., *p* = 0.237 for the S-warfarin/R-warfarin outcome). As previously reported (Niinuma et al., 2014), CYP2C9*9 had decreased ezyme activity towards S-warfarin with *p*-values being significant for S-warfarin/R-warfarin (*p* = 8.54 × 10^−8^, beta = 0.372) and S-6OH-warfarin/S-warfarin (*p* = 1.70 × 10^−4^, beta = −0.454). These results require further study as the histidine to arginine change at position 251 that defines this variant should have minimal effect on enzyme function (Pratt et al., 2019). Lastly, the *CYP2C* gene cluster variant rs12777823 (MAF 27.9%) was also replicated for S-warfarin/R-warfarin (*p* = 3.61 × 10^−8^) and S-7OH-warfarin/S-warfarin (*p* = 5.07 × 10^−7^). Although this variant has been previously associated with warfarin clearance in blacks (Perera et al., 2013), it is thought that this is because it is in linkage disequilibrium (LD) with an unknown causal variant as this effect has not been observed in other populations where it is common in the population (European MAF = 15.1%, East Asian MAF = 31.4%) (Genomes Project et al., 2010).

In the exploratory GWAS, 373 unique SNPs in 13 genes passed the Bonferroni-adjusted genome-wide significance threshold (*p* < 3.846 × 10^−9^), with most (*n* = 325, 87%) SNPs being associated with the S-warfarin/R-warfarin outcome. The functional relevance of more than a third (69%) of these SNPs could be due to LD (*r* ^2^ = 0.8) with the widely-known variants CYP2C9*8 (*n* = 216) and rs12777823 (*n* = 8). Two other biologically plausible loci were the *CYP2C9* 3′ untranslated region (UTR) SNP rs9332241 (*p*-value with S-warfarin/R-warfarin outcome = 5.38 × 10^−10^) and the *CYP2C18* stop-gained SNP rs41291550 (*p* = 3.70 × 10^−9^). The 3′ UTR SNP rs9332241 could play a role in gene silencing either by translational repression or by mRNA degradation through microRNA regulation. The stop-gained SNP rs41291550 was not associated with any literature in the National Library of Medicine’s National Centre for Biotechnological Information (NCBI) SNP database and its clinical significance is still unknown. It is mentioned in a cancer-related publication (Li et al., 2019), however, it is listed as non-cancer promoting and it is not linked to any disease. Additionally, the undetectable hepatic expression of CYP2C18 at protein level may mean this SNP plays little role in the metabolism of warfarin (Lofgren et al., 2008; Esteban et al., 2020).

Seventy-nine (21%) SNPs had statistically significant expression quantitative trait loci (eQTLs) with eight SNPs potentially regulating *CYP2C19* hepatic expression. However, and compared to *CYP2C9* (metabolises the more potent warfarin enationer), *CYP2C19* is less important in warfarin’s metabolism as it metabolises the less potent counterpart (R-warfarin) into minor metabolites (R-6-OH-warfarin and R-8-OH-warfarin, Supplementary Figure S1). Lastly, 75 (20%) SNPs had statistically significant splicing quantitative trait loci (sQTLs), including some significantly associated with the hepatic splicing of *CYP2C8* (*n* = 30), *CYP2C18* (*n* = 8), *CYP2C19* (*n* = 8) and *CYP2C9* (*n* = 4) mRNAs. Alternative Splicing of these cytochrome P450 mRNAs can result in protein isoforms with altered function including lack of enzymatic activity (Ariyoshi et al., 2007; Annalora et al., 2017). Since the liver is the primary metabolism site for warfarin, these hepatic eQTLs and sQTLs are biologically plausible loci. Further study (external replication and functional characterization) of these SNPs is required for full understanding.

Our study has limitations. For SNPs with low MAFs, our sample size was not large enough (despite having recruited over 500 patients) to replicate some previously reported associations such as with CYP2C9*3, CYP2C9*5 and CYP2C9*6. As explained in the introduction, recruiting participants from areas where pharmacogenomic-related clinical research is in its early infancy is challenging (Teo et al., 2010) and this is complicated by the extreme genetic diversity of African populations (Tishkoff et al., 2009) which necessitates very large sample sizes. Given the paucity of pharmacogenomic evidence in sub-Saharan Africa, studies like ours are important since they help stimulate further studies through the setting up of clinical research infrastructure and changing researcher/clinician/patient perspectives of pharmacogenomic research. Additionally, results from studies such as ours can be combined with future studies through meta-analysis. Due to high correlation between some of the study end-points (Supplementary Figure S4), we may have used a very strict genome-wide significant threshold (*p* < 3.846 × 10^−9^) by applying a Bonferroni adjustment. However, we have also reported all nominally-significant (*p* < 1 × 10^−5^) SNPs, and these include all the signals that our strict threshold may have missed. We did not measure the time between the last dose and blood sampling. However, warfarin is usually given in the evenings and INR readings are usually taken in the morning for the majority of patients. We also included patients on stable warfarin dose, which means that they had reached steady state and together with the long half-life of warfarin (between 20 and 60 h) meant that the concentrations of the parent compounds (and metabolites) were more or less consistent. Additionally, fluctuating concentrations were less relevant to the ratio outcomes, including the S-warfarin/R-warfarin outcome, which produced the most significant hits. Another limitation is that we only included patients from Uganda and South Africa which means our results may not be generalizable to the rest of sub-Saharan Africa given the very high within-population genetic diversity of African populations (Tishkoff et al., 2009). Our cohort also excluded patients who had not yet achieved a stable warfarin dose, and children, which limits generalizability to these population categories. Finally, we do not have a replication cohort for the GWAS hits outside the *CYP2C* gene cluster, and thus our findings are exploratory in nature.

In conclusion, we have provided further evidence to confirm the role of *CYP2C9* in warfarin dosing and shown that variants other than CYP2C9*2 and CYP2C9*3 are more important in sub-Saharan black-Africans. In exploratory work, we have conducted the first warfarin pharmacokinetics-related GWAS in sub-Saharan African populations from Uganda and South Africa, and identified novel SNPs that will require external replication and functional characterization before they can be clinically-implemented. We have used a pharmacokinetic approach as it is more powerful compared to the commonly-used stable dose approach. A stable dose GWAS is nevertheless planned and will have a larger sample size to take into account the genetic diversity in the region, and will include patients of mixed-ancestry.

## Data Availability

The derived meta-analysis summary statistics generated in this study have been deposited in the GWAS catalogue under accession code GCP000391 (https://www.ebi.ac.uk/gwas/). The accession numbers for each of the study endpoints are found in Supplementary Table S24.
