# Supplementary material for: A genome-wide association study of plasma concentrations of warfarin enantiomers and metabolites in sub-Saharan black-African patients
Source: Front Pharmacol. 2022 Sep 23;13:967082. doi: 10.3389/fphar.2022.967082 (PMC9537548; doi:10.3389/fphar.2022.967082)
Supplement: Supplementary file 1 [file DataSheet1.zip › GWAS_PK_supplementary_text_and_figures.docx]

**A** **genome-wide association study of plasma concentrations of warfarin enantiomers** **and metabolites in sub-Saharan Black-African patients.**

Innocent G. Asiimwe^1*^, Marc Blockman^2^, Karen Cohen^2^, Clint Cupido^3^, Claire Hutchinson^1^, Barry Jacobson^4^, Mohammed Lamorde^5^, Jennie Morgan^6^, Johannes P. Mouton^2^, Doreen Nakagaayi^7^, Emmy Okello^7^, Elise Schapkaitz^8^, Christine Sekaggya-Wiltshire^5^, Jerome R. Semakula^5^, Catriona Waitt^1,5^, Eunice J. Zhang^1^, Andrea L. Jorgensen^9,10^, and Munir Pirmohamed^1,10*^

^1^The Wolfson Centre for Personalized Medicine, Department of Pharmacology and Therapeutics, Institute of Systems, Molecular and Integrative Biology, University of Liverpool, United Kingdom. ^2^Division of Clinical Pharmacology, Department of Medicine, University of Cape Town, Cape Town, South Africa. ^3^Victoria Hospital Internal Medicine Research Initiative, Victoria Hospital Wynberg and Department of Medicine, University of Cape Town, Cape Town, South Africa. ^4^Department of Molecular Medicine and Haematology, University of the Witwatersrand, Johannesburg, South Africa. ^5^Infectious Diseases Institute, Makerere University College of Health Sciences, Kampala, Uganda. ^6^Metro District Health Services, Western Cape Department of Health, South Africa. ^7^Uganda Heart Institute, Kampala, Uganda. ^8^Department of Molecular Medicine and Hematology, Charlotte Maxeke Johannesburg Academic Hospital National Health Laboratory System Complex and University of Witwatersrand, Johannesburg, South Africa. ^9^Department of Health Data Science, Institute of Population Health Sciences, University of Liverpool, United Kingdom. ^10^These authors contributed equally.

***Correspondence**

Innocent G. Asiimwe

[i.asiimwe@liverpool.ac.uk](mailto:i.asiimwe@liverpool.ac.uk)

Munir Pirmohamed

[munirp@liverpool.ac.uk](mailto:munirp@liverpool.ac.uk)

**Table of contents**

**Supplementary Text………………………………………….……………………………………………………………2**

**Supplementary Figures………………………………………………………………….……………………………...17**

**Supplementary References……………………………………………………….……………………….………….24**

**Supplementary Text**

**Supplementary Text S1. Chromatographic solvents and buffers, sample preparation and data acquisition for the high-performance liquid chromatography-tandem mass spectrometry assay.**

Chromatographic solvents and buffers were: mobile phase A, water 0.1% formic acid; mobile phase B, methanol 0.1% formic acid; wash buffer, isopropanol 50%; rinse buffer, water 0.1% formic acid; extraction buffer, acetonitrile 1% formic acid spiked with warfarin-d5, 4′-OH-warfarin-d4, 7'-OH-warfarin-d5 and 8'-OH-warfarin-d5; and, diluent, 60% methanol 0.1% formic acid. All solvents/buffers were freshly-prepared (i.e. used within 7 days of preparation), of HPLC-MS/MS grade and purchased from Fisher Scientific (UK). Standard/calibration curves for each isomer and metabolites were prepared using stock racemic compounds (warfarin, 4-OH-warfarin, 6-OH-warfarin, 7-OH-warfarin, 8-OH-warfarin, and 10-'OH-warfarin). Deuterated warfarin (S)-Warfarin-d5 and (R)-Warfarin-d5 served as internal standards (ISs) for the parent compounds, while IS for the metabolites were (S)-4-OH-warfarin-d4 for (S)-4-OH-warfarin, (R)-4-OH-warfarin-d4 for (R)-4-OH-warfarin and (RS)-10'-OH-warfarin, (S)-7-OH-warfarin-d5 for (S)-6-OH-warfarin and (S)-7-OH-warfarin, (R)-7-OH-warfarin-d5 for (R)-6-OH-warfarin and (R)-7-OH-warfarin, (S)-8-OH-warfarin-d5 for (S)-8-OH-warfarin and (R)-8-OH-warfarin-d5 for (R)-8-OH-warfarin. All compounds were purchased from Toronto Research Chemicals Inc (Canada).

Prior to HPLC-MS/MS analysis, frozen K3-EDTA plasma samples acquired during patient enrolment and received from collaborators in South Africa and Uganda were defrosted followed by heat treatment at 58°C for 45 minutes in a biosafety level 3 laboratory (University of Liverpool, UK) to inactivate any viable human immunodeficiency virus present. Plasma samples from TB positive participants and those with unknown TB status required an additional filtration step using a 0.1 µm syringe filter to remove any viable Mycobacterium tuberculosis before heat inactivation. Inactivated plasma samples were then transported to a biosafety level 2 laboratory (University of Liverpool, UK) for downstream work.

To remove precipitates in the inactivated plasma, samples were centrifuged at 21,000 x g at 4°C for an average of 15 minutes. Protein precipitation and phospholipid removal was then carried out by adding 50 µL plasma into an Ostro plate (Waters Corp, USA) attached to a 96-well deep-well collection plate, followed by 450 µL extraction buffer containing deuterated ISs. Thorough mixing by shaking at 600 rpm (PMS-1000i Microplate shaker, Grant Instruments (Cambridge) Ltd, UK) for 10 minutes was essential before centrifugation at 500 x g for 5 minutes. The deep-well collection plate containing the extracted plasma was thereafter dried at 48°C using a nitrogen-piped evaporator. After evaporation, each well was reconstituted with 50 µL of diluent and placed in the autosampler at 4°C within the Shimadzu Nexera X2 modular system (Shimadzu, UK). A 5 μL aliquot was first injected into a 5 µm achiral Kinetex F5 column (Kinetex F5 100 Å (100 x 3.0 mm ID, 100Å); Phenomenex Ltd, UK), before passing into a 3 µm chiral Lux Cellulose-1 column (100 x 3.0 mm ID; Phenomenex Ltd, UK) at a flow rate of 0.5 mL/min, with both columns heated to 40°C. Analytes were separated via gradient elution (parameters detailed in Supplementary Table 3).

Data acquisition was carried out using multiple reaction monitoring (MRM, MS/MS) in the low mass setting with a Turbo V™ electrospray source operated in positive ionisation mode within the Sciex triple quadrupole 6500 QTRAP mass spectrometer (AB Sciex, Warrington, UK). The specific MRM transitions (*m/z*) used for quantification were: warfarin 308.9 → 251.2, 4’-, 6-, 7-, 8-OH warfarin 324.8 → 267.0, 10-OH warfarin 324.8 → 251, warfarin-d5 308.9 → 251.2, 4-OH warfarin-d4 329.2 → 271.3, 7-OH warfarin-d5 330.3 → 271.8, and 8-OH warfarin-d5 330.1 → 272.2.

**Supplementary Text S2. Quality Control (QC) and analysis protocol**

Raw data was generated using the Illumina Infinium H3Africa Consortium Array v2 (2,271,503 SNPs) as .ped and .map PLINK files. These were converted to binary PLINK file formats (.bed, .bim and .fam, using the --make-bed command) and uploaded on the bioinf1 cluster (pathnames shown below).

WARPATH binary PLINK file:

/ph-users/shared/Eunice/WARPATH/WARPATH.bed

/ph-users/shared/Eunice/WARPATH/WARPATH.bim

/ph-users/shared/Eunice/WARPATH/WARPATH.fam

Adding sex information

Opened up the fam file in Excel. Kept Family ID (‘FID’) and Within-family ID (‘IID’). Based on clinical data, column E was changed to ‘1’ for males, ‘2’ for females, and ‘0’ for unknown. Columns C, D and F were deleted, the new file saved as a text file (WARPATH_gender.txt) and used to create an updated fam file using the command below.

/ph-users/shared/Eunice/For_Maria/plink1.9/plink --bfile /ph-users/shared/Eunice/WARPATH/WARPATH --update-sex WARPATH_gender.txt --make-bed --out WARPATH_gender

#Above code was saved in a script (Update_gender.sh) and uploaded using the command: script_submit /ph-users/innocent/Update_gender.sh

**A: Per-sample QC**

1) Sex check

Generated a .sexcheck file using the following command:

/ph-users/shared/Eunice/For_Maria/plink1.9/plink --bfile WARPATH_gender --check-sex --out WARPATH_gender_sex_check

Produced a text file of samples with discordant sex using the command:

grep PROBLEM WARPATH_gender_sex_check.sexcheck > failed_sex_check_WARPATH.txt

#failed_sex_check_WARPATH.txt contains the first list of samples to be excluded (1 failed)

2) Checks for missing data

Used the following command:

/ph-users/shared/Eunice/For_Maria/plink1.9/plink --bfile WARPATH_gender --missing --out WARPATH_missing

To produce 2 files (.imiss and .lmiss):

a) a ‘.imiss’ file, summarises the proportion of missing genotype data per individual, and,

b) a ‘.lmiss’ file, summarises the proportion of missing genotype data per SNP

The fourth column in the imiss file (N_MISS) gives the number of missing SNPs and the

sixth column (F_MISS) gives the proportion of missing SNPs per individual.

#Total genotyping rate was 0.993257

An R_script (Missing.R) was used to produce a plot (shown below) showing the proportion of missing data per individual.

CR=read.table("WARPATH_missing.imiss",header=T)

pdf("WARPATH_missing_persample.pdf")

hist(CR$F_MISS,xlab="Proportion missing per individual",breaks=50)

dev.off()

The list of individuals to exclude based on a 5% threshold were obtained by:

awk '$6 >= 0.05 {print} WARPATH_missing.imiss > WARPATH_missing_remove

To know the number of excluded samples, typed:

awk 'NR>1 && $6 >= 0.05 {print}' WARPATH_missing.imiss | wc -l

3) Outlying heterozygosity

For heterozygosity checks, the following in PLINK command was run:

/ph-users/shared/Eunice/For_Maria/plink1.9/plink --bfile WARPATH_gender --het --out WARPATH_het

To identify outlying heterozygosity, a plot of mean heterozygosity versus proportion missing genotypes was ploted using the R script below.

CR=read.table("WARPATH_missing.imiss",header=T)

HET=read.table("WARPATH_het.het",header=T, as.is=T)

H=((HET$N.NM.-HET$O.HOM.)/HET$N.NM.)

pdf("WARPATH.Het_vs_imiss.pdf")

dat = data.frame(x=H, y=CR$F_MISS)

plot(dat,log="x",xlim=c(0.24,0.28),ylab="Fraction of missing genotypes",xlab="Mean

Heterozygosity")

abline(v=mean(H)-(2*sd(H)),col="BLUE",lty=2)

abline(v=mean(H)+(2*sd(H)),col="BLUE",lty=2)

abline(v=mean(H)-(3*sd(H)),col="GREEN",lty=2)

abline(v=mean(H)+(3*sd(H)),col="GREEN",lty=2)

abline(v=mean(H)-(5*sd(H)),col="RED",lty=2)

abline(v=mean(H)+(5*sd(H)),col="RED",lty=2)

abline(h=0.05,col="ORANGE",lty=2)

dev.off()

The WARPATH_het.het file (opened in Excel) was used to identify the outliers based on 5 sds.

4) Duplicate/related samples

Identity-by-state (IBS) and identity-by-descent (IBD) statistics for included patients helps to identify duplicated and/or related samples. This was done on a pruned subset of SNPs using the following command:

/ph-users/shared/Eunice/For_Maria/plink1.9/plink --bfile WARPATH_gender --indep-pairwise 50 5 0.2 --out WARPATH_pruned

The options 50, 5, and 0.2 mean that a window of 50 SNPs is considered, linkage disequilibrium (LD) is computed between each pair of SNPs in the window and one of a pair of SNPs with LD > 0.2 is removed before the window is shifted 5 SNPs forward and the procedure repeated.

Two files (.prune.in and .prune.out) are produced. Either can be used depending on which of PLINK’s ‘--extract’ or ‘--exclude’ commands is used. For this analysis, the ‘--extract’ command was used to produce a .genome file. (containing pair-wise IBS and IBD estimates for all pairs of patients based on the reduced marker set.) as shown below:

/ph-users/shared/Eunice/For_Maria/plink1.9/plink --bfile WARPATH_gender --extract WARPATH_pruned.prune.in --genome --out WARPATH_IBD

Used an IBD R script to produce an IBD histogram.

PI=read.table("WARPATH_IBD.genome",header=T,as.is=T)

pdf("WARPATH_ibd_histogram.pdf")

hist(PI$PI_HAT,ylim=c(0,100),xlim=c(0,1.0),main="WARPATH: IBD Estimation",xlab="Estimated mean pairwise IBD",ylab="Frequency")

dev.off()

To obtain a file that includes all outliers, the following command was used:

awk '$10 >=0.1875 {print}' WARPATH_IBD.genome > WARPATH_IBD_outliers

For the related samples, the one with the most amount of missingness (available in the F_MISS values from imiss file) was excluded. A .txt file of these individuals (IBS_outliers.txt) was manually created and used to subset the .imiss file using the R script below.

IMISS=read.table("WARPATH_missing.imiss",header=T)

Outlier=read.table("IBD_outliers.txt",header=T)

IMISS_IBD <- IMISS[IMISS$FID %in% Outlier$FID,]

write.table(IMISS_IBD, file = "IMISS_IBD.txt", sep = " ")

The related samples were compared and a list of samples with the most amount of missingness per pair generated (IBS_outliers_exclude.txt) for subsequent exclusion.

Four pairs had an IBD coefficient close to 1. Since it wasn’t certain which ID was correct (a clinician confirmed they were not related), we excluded all.

5) Ethnic outliers

To obtain the list of genotyped rs IDs (WARPATH_snp_list.txt), the WARPATH_gender.bim file was used, using the following command:

awk '{ print $2 }' WARPATH_gender.bim > WARPATH_snp_list.txt

These were merged with the 1000 genomes files (provided) using the following command (submitted as a script):

/ph-users/shared/Eunice/For_Maria/plink1.9/plink --bfile /ph-users/shared/Eunice/1000Genomes/PCA/EUR_EAS_AFR_1668pts --extract WARPATH_snp_list.txt --make-bed --out WARPATH_1000genomes

The list of SNPs in the above file were generated using:

awk '{ print $2 }' WARPATH_1000genomes.bim > WARPATH_1000_snp_list.txt

And merged with the WARPATH files:

/ph-users/shared/Eunice/For_Maria/plink1.9/plink --bfile WARPATH_gender --extract WARPATH_1000_snp_list.txt --make-bed --out WARPATH_1000

Both genome files were merged using the command:

/ph-users/shared/Eunice/For_Maria/plink1.9/plink --bfile WARPATH_1000genomes --bmerge WARPATH_1000.bed WARPATH_1000.bim WARPATH_1000.fam --make-bed --out WARPATH_1000_merged

A missnp file was created and so respective strands were flipped using:

/ph-users/shared/Eunice/For_Maria/plink1.9/plink --bfile WARPATH_1000genomes --flip WARPATH_1000_merged-merge.missnp --make-bed --out WARPATH_1000genomes_flipped

The merge then proceeded using the command:

/ph-users/shared/Eunice/For_Maria/plink1.9/plink --bfile WARPATH_1000genomes_flipped --bmerge WARPATH_1000.bed WARPATH_1000.bim WARPATH_1000.fam --make-bed --out WARPATH_1000_merged2

There were 97 variants with 3+ alleles present (WARPATH_1000_merged2-merge.missnp file) and so these were excluded from both genome files:

/ph-users/shared/Eunice/For_Maria/plink1.9/plink --bfile WARPATH_1000genomes --exclude WARPATH_1000_merged2-merge.missnp --make-bed --out WARPATH_1000genomes_exclude

/ph-users/shared/Eunice/For_Maria/plink1.9/plink --bfile WARPATH_1000 --exclude WARPATH_1000_merged2-merge.missnp --make-bed --out WARPATH_1000_exclude

Respective SNPs were flipped in the first file before the merge was re-attempted:

/ph-users/shared/Eunice/For_Maria/plink1.9/plink --bfile WARPATH_1000genomes_exclude --flip WARPATH_1000_merged-merge.missnp --make-bed --out WARPATH_1000genomes_exclude_flipped

/ph-users/shared/Eunice/For_Maria/plink1.9/plink --bfile WARPATH_1000genomes_exclude_flipped --bmerge WARPATH_1000_exclude.bed WARPATH_1000_exclude.bim WARPATH_1000_exclude.fam --make-bed --out WARPATH_1000_merged_final

A pruned subset was obtained as done for duplicate/related samples:

/ph-users/shared/Eunice/For_Maria/plink1.9/plink --bfile WARPATH_1000_merged_final --indep-pairwise 50 5 0.2 --out WARPATH_1000_merged_final_pruned

And a .genome file obtained similarly:

/ph-users/shared/Eunice/For_Maria/plink1.9/plink --bfile WARPATH_1000_merged_final --extract WARPATH_1000_merged_final_pruned.prune.in --genome --out WARPATH_ethnicity

A .mds file (containing the first 10 principal components) was generated using the command:

/ph-users/shared/Eunice/For_Maria/plink1.9/plink --bfile WARPATH_1000_merged_final --cluster --mds-plot 10 --read-genome WARPATH_ethnicity.genome --out WARPATH_MDS10

The .mds file was opened up in excel and ethnicities added (used /ph-users/shared/Eunice/1000Genomes/PCA/pop_EUR_EAS_AFR_1668pts.txt) for the 1000 genomes populations – study sample ethnicities left blank. Thereafter the PCA plot was plotted in R, and used to identify outliers.

data <- read.csv(file.path("C:/Users/oliasiim/Documents/GWAS/PCA.csv"), header = TRUE)

AFR <- data[data$super_pop == 'AFR',c('C2', 'C1')]

EUR <- data[data$super_pop == 'EUR',c('C2', 'C1')]

EAS <- data[data$super_pop == 'EAS',c('C2', 'C1')]

pdf("PCA.pdf", width = 5, height = 5)

plot(data$C2, data$C1, xlab = "Principal component 2", ylab = "Principal component 1")

points(AFR, col = "red", pch = 19)

points(EUR, col = "green", pch = 19)

points(EAS, col = "blue", pch = 19)

dev.off()

Summary

Starting with 561 eligible patients, 1 was excluded due to ‘sex check’ QC. Two additional samples were excluded based on having more than 5% missing genotype data, no additional samples due to extreme heterozygosity, and 1 to being an Ethnic outlier. Lastly 9 samples were excluded due to being related to already included samples/uncertainty in the phenotype data. This gave total of 13 excluded samples (or a total of 548 included samples).

A .txt file consisting of the 13 samples was made and incorporated into the command below to exclude samples from subsequent analysis.

/ph-users/shared/Eunice/For_Maria/plink1.9/plink --bfile WARPATH_gender --remove Samples_to_exclude.txt --make-bed --out WARPATH_clean

**B: Per-SNP QC**

1) Checks for missing data

This is similar to per-sample QC except that the .Imiss file is used:

/ph-users/shared/Eunice/For_Maria/plink1.9/plink --bfile WARPATH_clean --missing --out WARPATH_clean_missing

The R_script (Missing_SNP.R) to produce a plot was:

CR=read.table("WARPATH_clean_missing.lmiss",header=T)

pdf("WARPATH_missing_perSNP.pdf")

hist(CR$F_MISS,xlab="Proportion missing per SNP",breaks=50)

dev.off()

A 5% threshold was pre-specified. To know how many SNPs would be excluded, the following command was used:

awk 'NR>1 && $5 >= 0.05 {print}' WARPATH_clean_missing.lmiss | wc -l

2) Checks for MAF

To investigate how many SNPs have MAF <1%, we ran the following commands

/ph-users/shared/Eunice/For_Maria/plink1.9/plink --bfile WARPATH_clean --freq --out WARPATH_clean_MAF

awk '$5<0.01' WARPATH_clean_MAF.frq | wc -l

2) Checks for adherence to HWE

To generate a list of genotype counts and HWE test statistics for each SNP, the following commands were used:

/ph-users/shared/Eunice/For_Maria/plink1.9/plink --bfile WARPATH_clean --hardy --out WARPATH_clean_HWE

Several thresholds were tested as shown below:

awk '$9<0.0001' WARPATH_clean_HWE.hwe | wc -l

awk '$9<0.00001' WARPATH_clean_HWE.hwe | wc -l

awk '$9<0.000001' WARPATH_clean_HWE.hwe | wc -l

Summary

To select for SNPs with genotyping rate >95%, MAF > 0.01, and Hardy-Weinberg p-value >0.000001 using the following command:

/ph-users/shared/Eunice/For_Maria/plink1.9/plink --bfile WARPATH_clean --geno 0.05 --maf 0.01 --hwe 0.000001 --make-bed --out WARPATH_QC

**C: Pre-imputation data preparation and QC**

1) Download tools and sites

These are available from <https://imputationserver.readthedocs.io/en/latest/prepare-your-data/>. For this analysis, the perl script to perform the pre-imputation data preparation had been previously downloaded (/ph-users/shared/Eunice/1000Genomes/preimputation/HRC-1000G-check-bim-v4.3.0/HRC-1000G-check-bim.pl)

2) Convert ped/map to bed

The files (WARPATH_QC) already in bed format

3) Create a frequency file

/ph-users/shared/Eunice/For_Maria/plink1.9/plink --freq --bfile WARPATH_QC --out WARPATH_freq

4) Execute script

The script checks the genetic variants against the reference panel i.e. also requires the 1000 Genomes phase III legend file for pre-imputation data preparation (/ph-users/shared/Eunice/1000Genomes/preimputation/1000GP_Phase3_combined.legend.gz)

perl /ph-users/shared/Eunice/1000Genomes/preimputation/HRC-1000G-check-bim-v4.3.0/HRC-1000G-check-bim.pl -b WARPATH_QC.bim -f WARPATH_freq.frq -r /ph-users/shared/Eunice/1000Genomes/preimputation/1000GP_Phase3_combined.legend.gz -g -p AFR

#Submitted the above as a .sh script (GWAS_perl.sh).

5) Perform SNP QC

The above script produced several .txt files and a PLINK2 script (Run-plink.sh, used to perform SNP QC and at the same time convert to .vcf format). Changed ‘plink’ to ‘/ph-users/shared/Eunice/For_Maria/plink1.9/plink’ to use the stable 1.9 version and submitted the script.

script_submit Run-plink.sh

Binary and .vcf files per chromosome produced (chr23 not included in analysis).

6) Use bgzip (/users/apps/htslib/htslib-1.3.2/bgzip) to zip each .vcf file (.vcf.gz files produced)

/users/apps/htslib/htslib-1.3.2/bgzip WARPATH_QC-updated-chr1.vcf

Repeat for chr2-22.

7) Upload the .vcf.gz files to the Michigan Imputation Server for imputation (<https://imputationserver.sph.umich.edu/index.html>)

Genotype Imputation (Minimac4) 1.5.7

Reference panel (1000G Phase 3 v5)

Input files (requires VCF files – uploaded .vcf.gz files)

Array Build (GRCh37/hg19)

Rsq Filter (off)

Phasing (Eaglev2.4)

Population (AFR)

Mode (Quality Control & Imputation)

Links to download imputation results, logs and quality control reports (allele-frequency correlation statistics, potential frequency mismatches and excluded/typed-only SNPs) as well as a password to open the results are provided once the imputation is complete.

**D: GWAS post-imputation steps**

1) Download tools

The tool (p7zip_16.02_src_all.tar.bz2) for unzipping is available at <https://sourceforge.net/projects/p7zip/?source=typ_redirect>. Downloaded this on my computer and copied it to my folder on the cluster.

To unzip the tool, used the command:

tar xvjf p7zip_16.02_src_all.tar.bz2

A folder p7zip_16.02 was created. Changed my working directory to this folder using:

cd p7zip_16.02

2) Install p7zip

Instructions for further installation obtained from: <https://www.linuxfromscratch.org/blfs/view/svn/general/p7zip.html>

Install p7zip by running the following commands:

make all3

To test the results, issue:

make test

Now, as the root user:

make DEST_HOME=/ph-users/innocent \

DEST_MAN=/ph-users/innocent/share/man \

DEST_SHARE_DOC=/ph-users/innocent/share/doc/p7zip-17.04 install

3) Extract files

Used the commands below (submited as 1 script i.e. script_extract.sh), with the password provided by the imputation server being PPyAFHnLip.g47y:

/ph-users/innocent/bin/7z x -pPPyAFHnLip.g47y /ph-users/innocent/chr_1.zip

Repeat for chr2-22.

4) QC - filtering SNPs with estimated R^2^ <0.3

.info.gz files are too large to view unzipped, so use the command ‘less chr1.info.gz’ if you want to view them (press ‘q’ to quit), to filter (submited as 1 script for all chromosomes):

gzip -dc chr1.info.gz | awk '$7<0.3 {print$1, $7}' > chr1_rsq_snps_toexclude.txt

Repeat for chr2-22.

5) Converting vcf to plink files

plink2_submit --vcf chr1.dose.vcf.gz --make-bed --out chr1_plink

Repeat for chr2-22.

6) Generating frequency files

/ph-users/shared/Eunice/For_Maria/plink1.9/plink --bfile chr1_plink --freq --out chr1_freq

Repeat for chr2-22.

7) Extracting SNPs with MAF <1%

awk '$5<0.01 {print$2, $5}' chr1_freq.frq > chr1_maf_toexclude.txt

Repeat for chr2-22.

8) Combining SNPs to exclude (based on R^2^ and MAF)

awk '{print $1}' chr1_rsq_snps_toexclude.txt < (awk '{print $1}' chr1_maf_toexclude.txt) > chr1_excludesnps.txt

Repeat for chr2-22.

9) Excluding SNPs from original .vcf file

Submit .sh scripts for each chr using the command:

/ph-users/shared/Eunice/vcftools_0.1.13/bin/vcftools --gzvcf chr1.dose.vcf.gz --exclude chr1_excludesnps.txt --recode --stdout

Before submitting the next script/command, submit the command below – important because stdout is overwritten by the next command:

/users/apps/htslib/htslib-1.3.2/bgzip -c stdout > chr1_qc_bgzip.vcf.gz

Repeat for chr2-22.

**E: Statistical analysis**

1) Multiple linear regression (each SNP with 14 covariates)

11 (2% of 548) patients were missing weight information. This was imputed using single imputation in MICE (based on country of recruitment, age, weight, gender, target INR, HIV status, simvastatin/amiodarone status, ten principal components and weekly dose).

A ‘WARPATH_covariate.sample’ file (space separated) was created. It comprised of three parts:

1. A header line detailing the names of the file columns (ID_1, ID_2, missing (missing data proportion of each individual), Age_years, Weight_kg, GenderMale, Simvastatin_amiodaroneYes, EfavirenzYes, C1, C2, C3, C4, C5, C6, C7, C8. C9, C10 and the different endpoints/phenotypes) - phenotypes should appear after the covariates.
2. A line detailing the variable types in each column (first 3 entries set to 0, discrete variables (coded using positive integers) set to D, continuous covariates to C and continuous phenotype to P).
3. A line for each individual detailing the individual’s information. Individuals with missing values for covariates and phenotypes (none in our case) should be coded -9 or NA.

For each of the outcomes, the following command was used (SNPtest.sh, note that for -frequentist specifies the genetic mode of inheritance i.e. 1=Additive; 2=Dominant; 3=Recessive; 4=General (i.e. no mode assumed)):

Make separate scripts per chromosome:

snptest -data chr1_qc_bgzip.vcf.gz WARPATH_covariate_PK.sample -pheno Rwarfarin -frequentist 1 -method expected -cov_names Age_years Weight_kg GenderMale Simvastatin_amiodaroneYes C1 C2 C3 C4 C5 C6 C7 C8 C9 C10 -o WARPATH_Swarfarin_chr1_add.out

Repeat for chr2-22.

The first 11 and last lines are not necessary. The 12^th^ line (column names) was also deleted since we will be concatenating the files. These were deleted using the following commands:

sed '1,12d;$d' WARPATH_chr1_add.out > chr001.txt

Repeat for chr2-22.

To concatenate all the above files, submitted a script (concat_chr.sh) that contained the command:

cat chr0*.txt > chr1_22.txt

To the above script, also added the following commands (to select only the columns of interest, label them, and remove any ‘NA’ p-values):

echo "SNP CHR BP P alleleA alleleB all_total all_maf frequentist_add_beta_1 frequentist_add_se_1" > WARPATH_Manhattan_temp.txt

awk '{print $2,$3,$4,$21,$5,$6,$18,$19,$23,$24}' chr1_22.txt >> WARPATH_Manhattan_temp.txt

sed '/NA/d' WARPATH_Manhattan_temp.txt > WARPATH_Manhattan.txt

A Manhattan plot was obtained using the following R script (Manhattan.R):

library(qqman)

library(data.table)

results= fread("WARPATH_Manhattan.txt")

png("WARPATH_Swarfarin_Manhattan.png", width=1500, height=800, res=120)

manhattan(results, main = "Manhattan plot: linear regression", genomewideline = -log10(5e-8/13), ylim = c(0, 10), col = c("blue4", "orange3"))

dev.off()

#For QQplot

png("WARPATH_Swarfarin_qqplot.png", width=1500, height=1500, res=120)

qq(results$P, main = "QQ plot: linear regression", col = "blue4")

dev.off()

#To calculate the genomic inflation factor, lambda,

chisq = qchisq(results$P,1,lower.tail=FALSE)

lambda <- median(chisq) / qchisq(0.5,1)

lambda

2) Getting rsIDs for the top SNPs from 1000 genomes reference panel based on position

grep -E -w '25917488|37578941|…|29606907|29608615' /ph-users/shared/Eunice/1000Genomes/PCA/EUR_EAS_AFR_1668pts.bim > topsnps_rs_ids.txt

3) Determining imputation status for the top SNPs

gzip -dc chr1.info.gz | grep -E -w '1:25917488:G:A|1:37578941:T:C|…|22:29606907:C:T|22:29608615:C:T' > imp_status_chr001.txt

Repeat for chr2-22.

4) Generating regional plots using LocusZoom

To zoom in, we used <http://locuszoom.org/> and selected ‘Single Plot Your data – original locuszoom)

• Uploaded (Path to your File) ‘WARPATH_QC_snptest_add2.out’ (ensure rsIDs are included)

• Changed P-Value Column Name to ‘frequentist_add_pvalue’

• Changed Marker Column Name to ‘rsid’

• Changed Column Delimiter to ‘WhiteSpace’

• Added SNP name (use the same for title)

• For genome build, left the selected option as hg19/1000 Genomes Nov 2014 AFR

• Clicked plot data.

• A pdf of the graph was automatically downloaded from the server.

**Supplementary Figures**


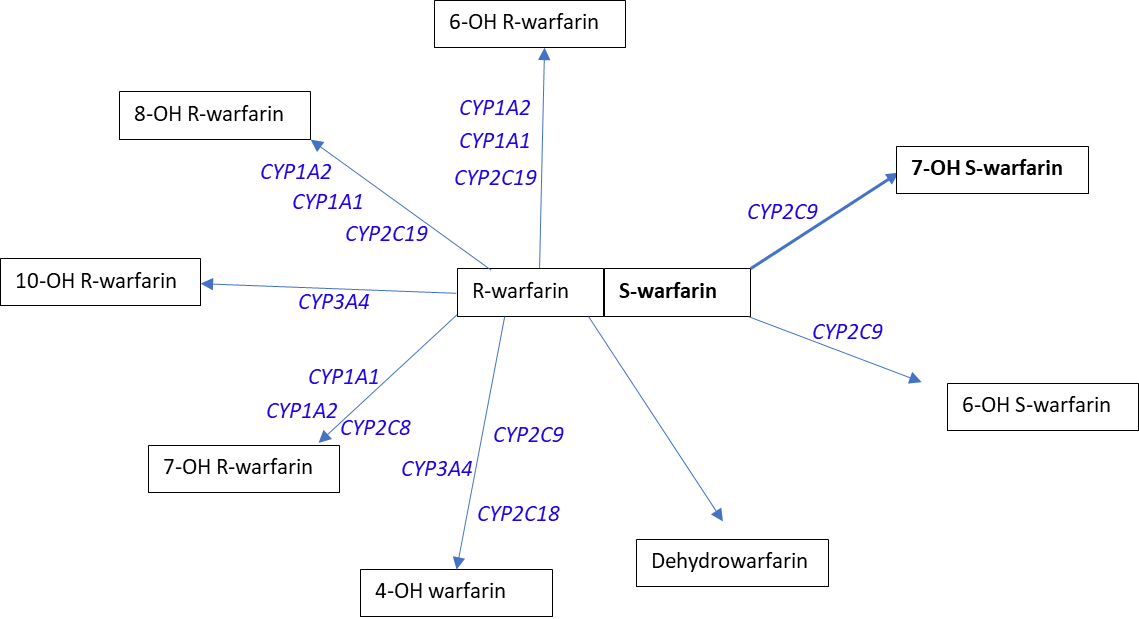


**Supplementary Figure S1. Warfarin pharmacokinetics pathway.** Warfarin is given as a racemic mixture, (R)- and (S)-stereoisomers, with the (S)-isomer being 3 to 5 times more potent. The more potent (S)-isomer is mainly metabolized by *CYP2C9* to 7-OH warfarin (main metabolite) and 6-OH warfarin. CYP = cytochrome P450. Adapted from the Pharmacogenomics Knowledge Base (Whirl-Carrillo et al., 2012).


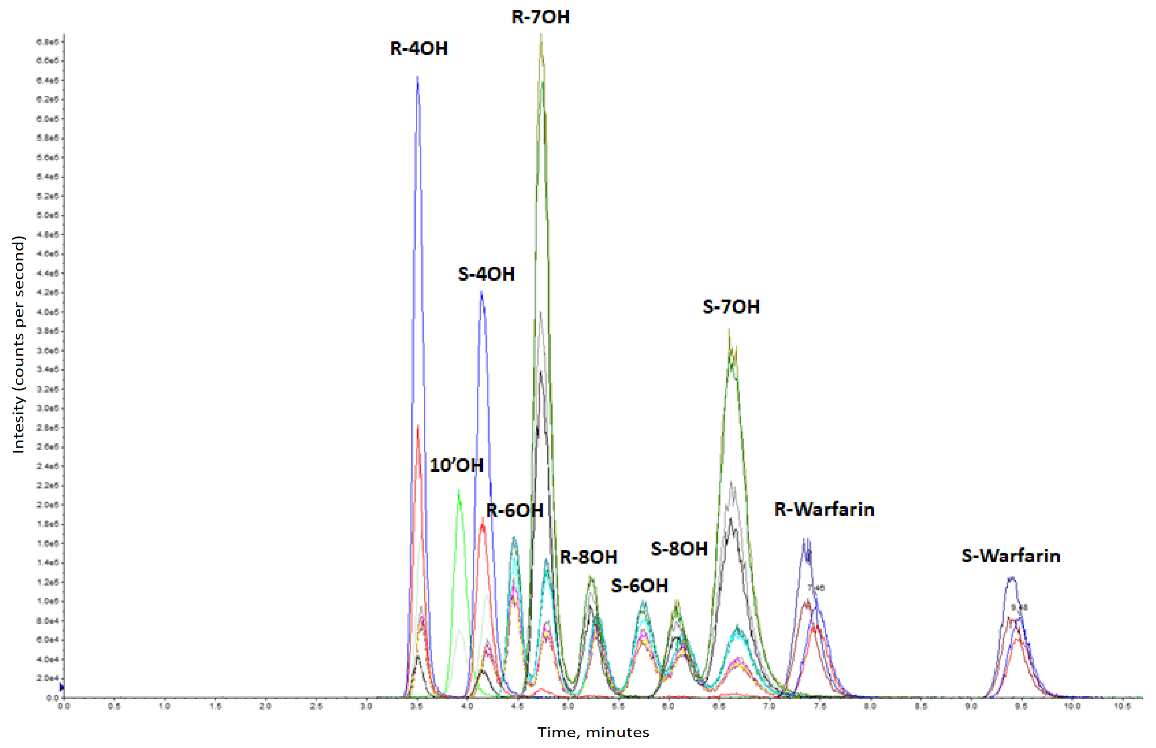


**Supplementary Figure S2. Chromatogram or (R)/(S)-warfarin and corresponding metabolites.**


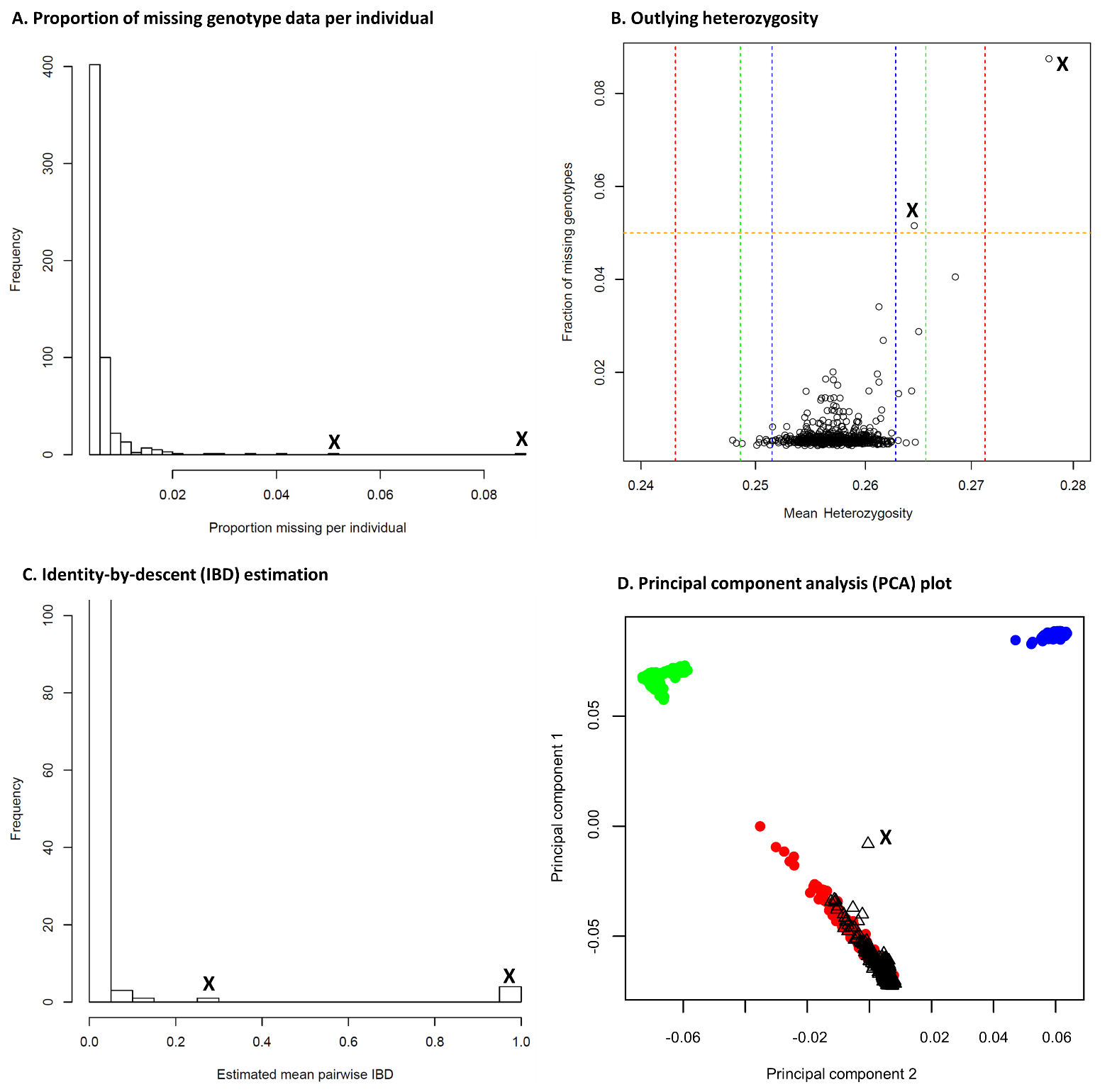


**Supplementary Figure S****3. Per-sample/individual quality control checks.** ‘X’ marks the individuals who were excluded. Two individuals were excluded due to having >5% missing genotype data (**panel A**). These same individuals are shown in **panel B** (above the horizontal orange dashed line which indicates a 95% genotype call rate threshold). One of these 2 individuals also had a heterozygosity rate greater than 5 standard deviations above the mean heterozygosity rate (blue, green and red vertical lines respectively represent thresholds of 2, 3 and 5 standard deviations). Regarding the identity-by-descent (IBD) estimate (**panel C**), 10 individuals (5 pairs) had an IBD coefficient >0.1875. From one pair (just above an IBD of 0.2), the individual with the most missing amount of genotype data was excluded. From four other pairs (close to an IBD of 1), all 8 individuals were excluded since an IBD of 1 (monozygotic twins/duplicated sample) was inconsistent with clinical information (it was confirmed that these individuals were different and none of them were twins). Lastly, 1 individual was excluded due to failure to cluster with the 1000 genomes African populations (**panel D**, colour coding: study samples = open triangles, Africans (1000 genomes) = red circles, Europeans (1000 genomes) = green circles, and East Asians (1000 genomes) = blue circles). The individual who was excluded due to discordant clinical/X-chromosome-derived gender not shown.

**
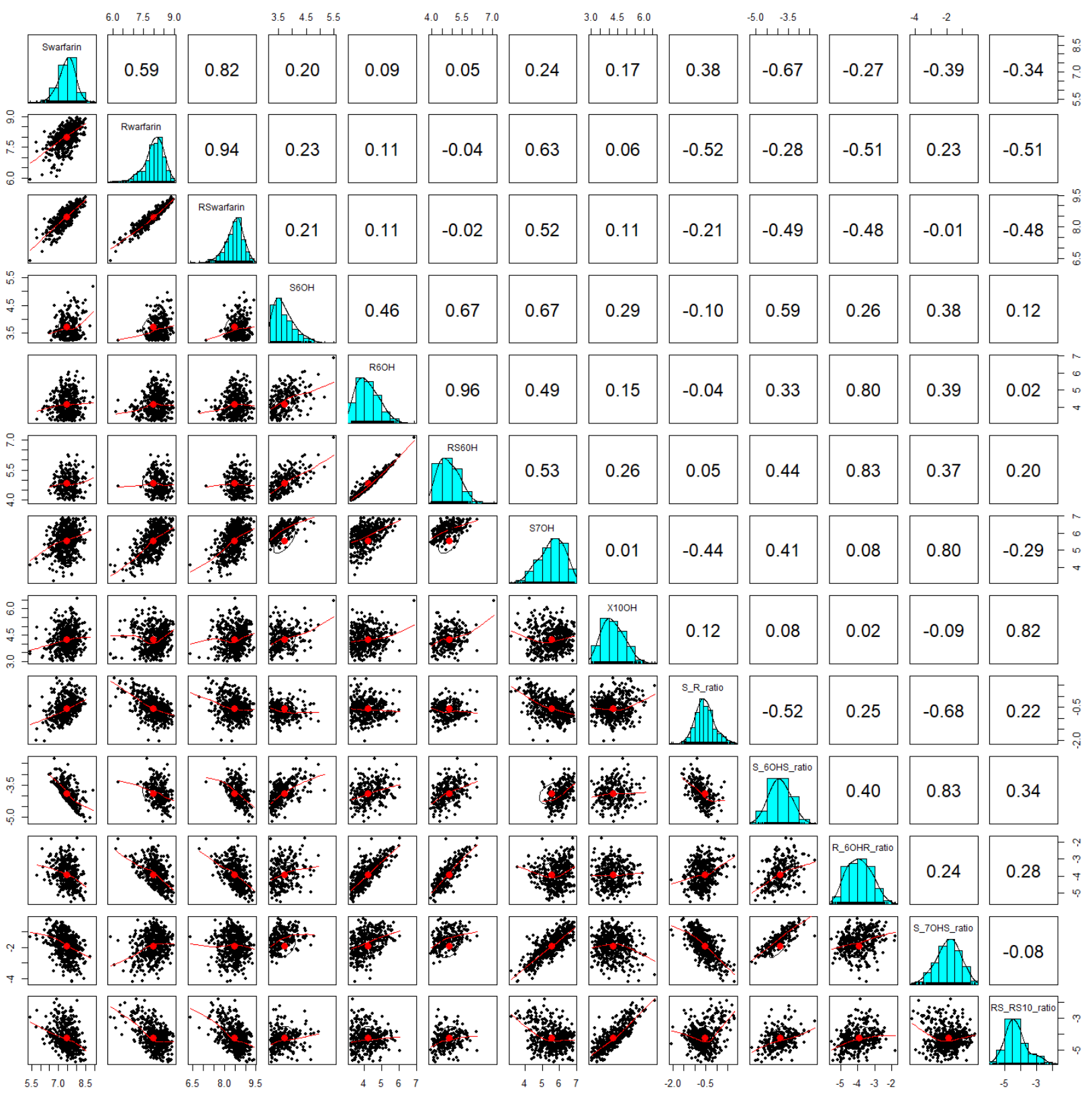
**

**Supplementary Figure S4. An enhanced scatterplot matrix showing pairwise correlations between the outcomes.** This figure was created with the pairs.panels() function in the R psych package (Revelle, 2020)). On the diagonal, histograms depicting the respective distributions are shown. Correlation coefficients are shown above the diagonal while scatterplots are shown below it. In the scatter plots, the oval-shaped black object is the correlation ellipse which reflects how strongly correlated the variables are, with the red dot at the centre representing the point of the mean values for the x and y axis variables and the ellipse shape indicating the correlation between the variables (a perfectly round oval represents a very weak correlation). The loess curve (red curve on the scatter plots) indicates the general relationship between the x and y axis variables with linear relationships having linear curves and non-linear relationships having non-linear curves.

**
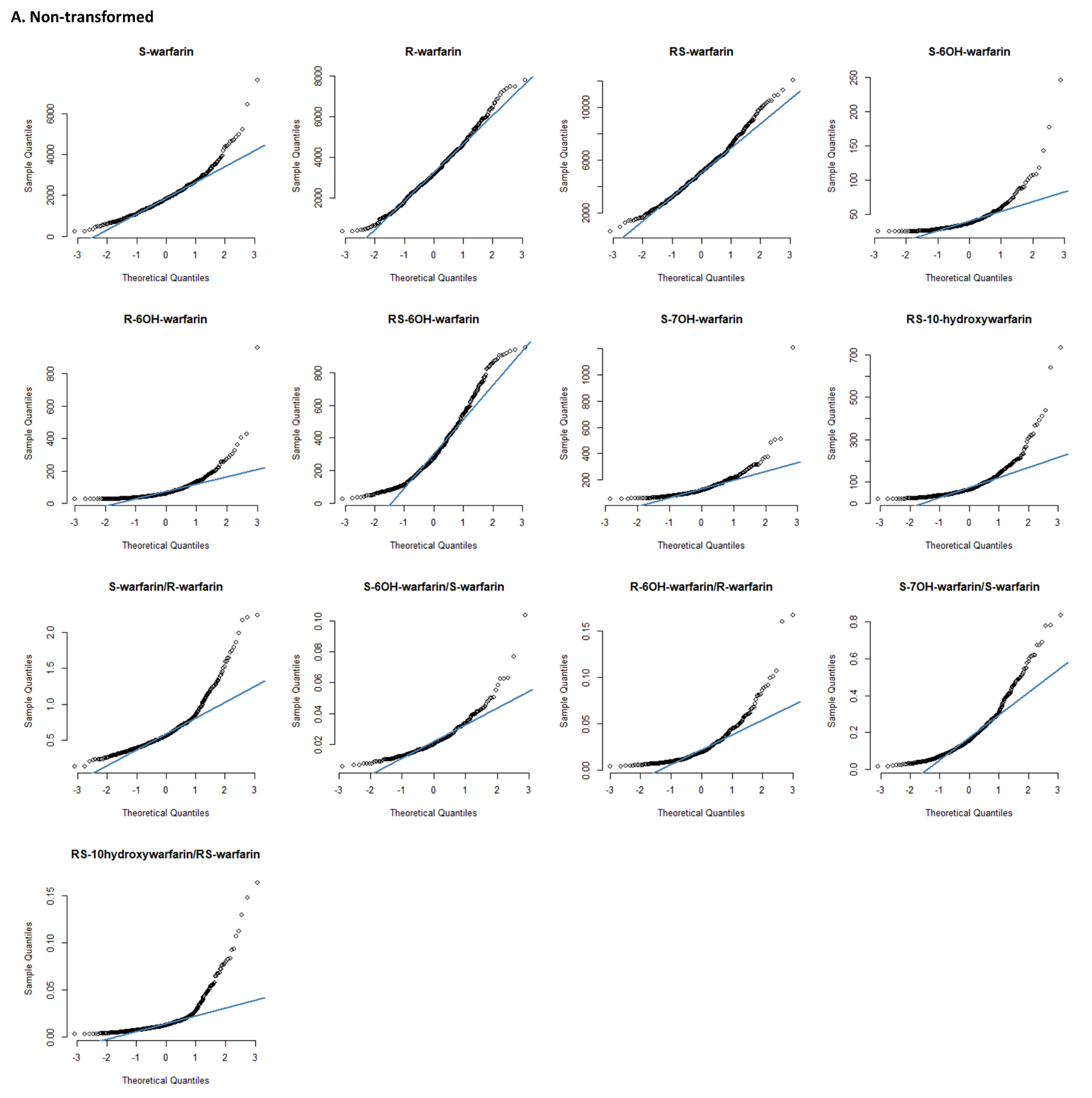
**

**Supplementary Figure S5. QQ** **plots before and after applying a logarithmic transformation to the study end-points.** QQ = quantile–quantile.

**
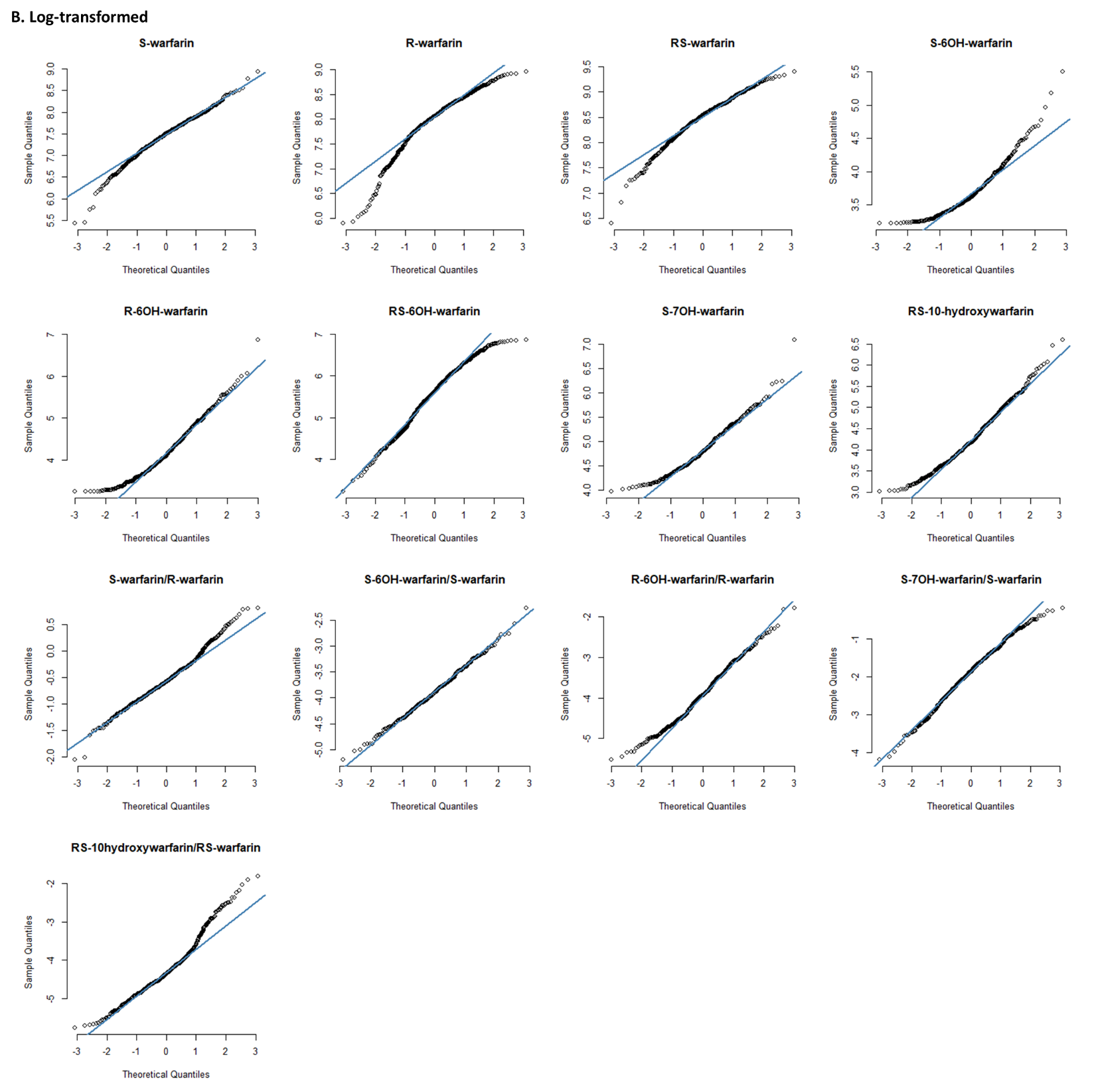
**

**Supplementary Figure S5. Continued.**

**
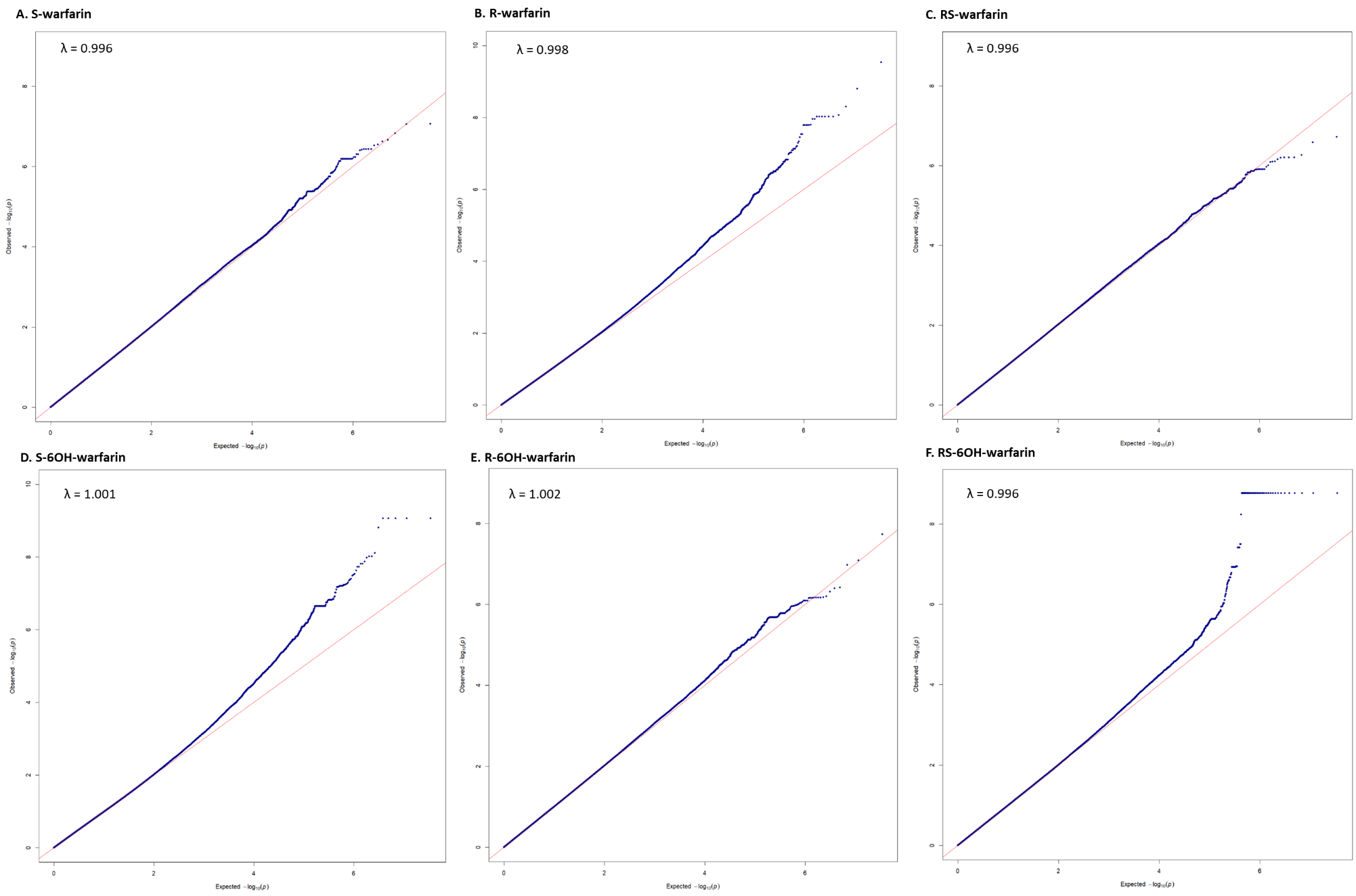
**

**Supplementary Figure S6. QQ plot of the association between SNPs and warfarin enantiomers, metabolites and analyte ratios.** QQ = quantile–quantile, λ = lambda (genomic inflation factor).

**
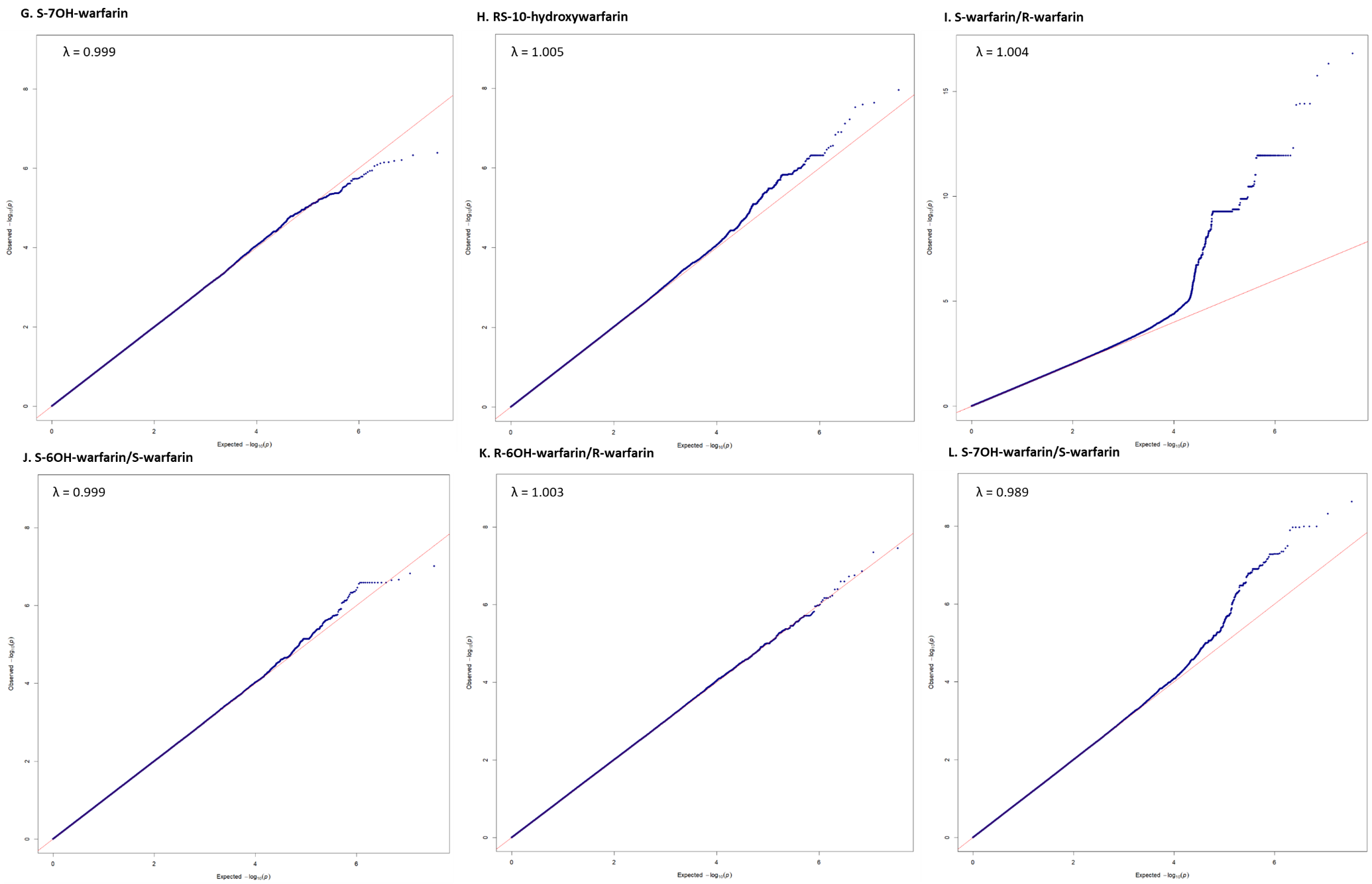
**

**Supplementary Figure S6. Continued.**

**
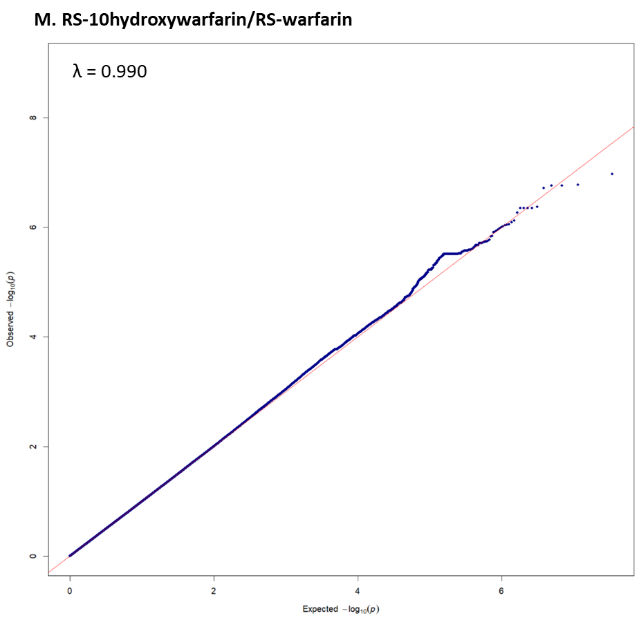
**

**Supplementary Figure S6. Continued.**

**Supplementary References**

Revelle, W. (2020). "psych: Procedures for Psychological, Psychometric, and Personality Research". R package version 2.1.3 ed. (Evanston, Illinois: Northwestern University).

Whirl-Carrillo, M., Mcdonagh, E.M., Hebert, J.M., Gong, L., Sangkuhl, K., Thorn, C.F., Altman, R.B., and Klein, T.E. (2012). Pharmacogenomics knowledge for personalized medicine. *Clin Pharmacol Ther* 92**,** 414-417.
